# An efficient multi-level thresholding method for breast thermograms analysis based on an improved BWO algorithm

**DOI:** 10.1186/s12880-024-01361-x

**Published:** 2024-07-30

**Authors:** Simrandeep Singh, Harbinder Singh, Nitin Mittal, Supreet Singh, S. S. Askar, Ahmad M. Alshamrani, Mohamed Abouhawwash

**Affiliations:** 1 Department of Electronics & Communication Engineering, UCRD, Chandigarh University, Gharuan, Punjab India; 2 VISILAB, Universidad de Castilla-La Mancha, Ciudad Real, 13071 Spain; 3 Skill Faculty of Engineering and Technology, Shri Vishwakarma Skill University, Palwal, 121102 India; 4 School of Computer Science, UPES, Dehradun, Uttarakhand India; 5 Department of Statistics and Operations Research, College of Science, King Saud University, P.O. Box 2455, Riyadh, 11451 Saudi Arabia; 6 Department of Mathematics, Faculty of Science, Mansoura University, Mansoura, 35516 Egypt

**Keywords:** Thresholding, IBWOA, Breast cancer, Thermography, Otsu, Kapur’s entropy

## Abstract

Breast cancer is a prevalent disease and the second leading cause of death in women globally. Various imaging techniques, including mammography, ultrasonography, X-ray, and magnetic resonance, are employed for detection. Thermography shows significant promise for early breast disease detection, offering advantages such as being non-ionizing, non-invasive, cost-effective, and providing real-time results. Medical image segmentation is crucial in image analysis, and this study introduces a thermographic image segmentation algorithm using the improved Black Widow Optimization Algorithm (IBWOA). While the standard BWOA is effective for complex optimization problems, it has issues with stagnation and balancing exploration and exploitation. The proposed method enhances exploration with Levy flights and improves exploitation with quasi-opposition-based learning. Comparing IBWOA with other algorithms like Harris Hawks Optimization (HHO), Linear Success-History based Adaptive Differential Evolution (LSHADE), and the whale optimization algorithm (WOA), sine cosine algorithm (SCA), and black widow optimization (BWO) using otsu and Kapur's entropy method. Results show IBWOA delivers superior performance in both qualitative and quantitative analyses including visual inspection and metrics such as fitness value, threshold values, peak signal-to-noise ratio (PSNR), structural similarity index measure (SSIM), and feature similarity index (FSIM). Experimental results demonstrate the outperformance of the proposed IBWOA, validating its effectiveness and superiority.

## Introduction

**Fig. 1:**
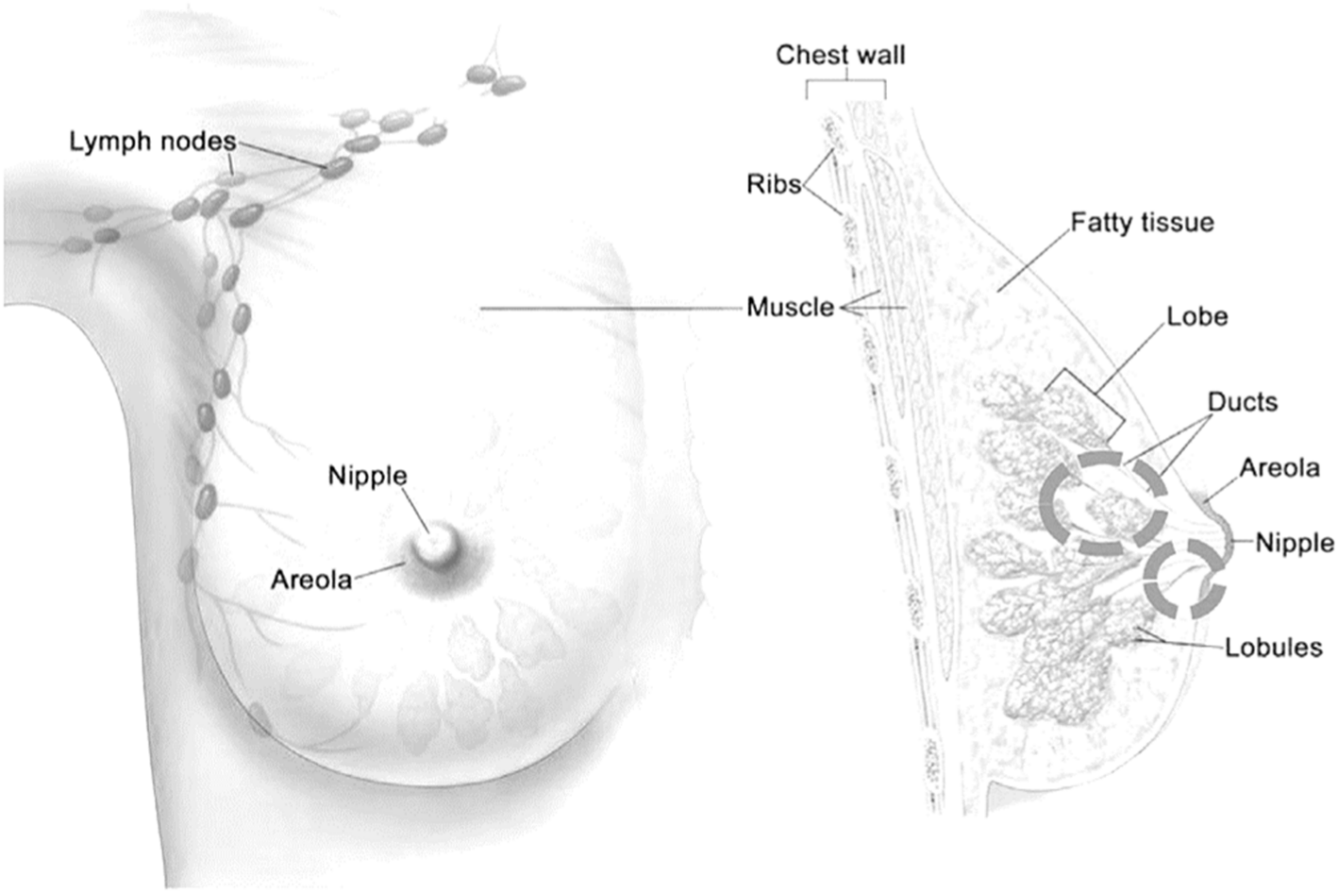
Most sensitive area for malignant tissue [5]

According to the World Health Organization, Breast cancer is the second most common cancer among women worldwide after lung cancer. Breast cancer accounted for more than five hundred thousand deaths each year and 1.7 million new cases are identified every year [1]. Breast cancer is one of the most often diagnosed tumor forms across the globe [2]. As a result of this exponential expansion, there is a substantial advancement in the field of novel technologies for early detection and prevention of breast cancer. If breast cancer is treated promptly, it can be healed, and also the death rate can be lowered. As a result, early detection is critical for enhancing survivorship, and frequent checks are required for those who may be at risk. It is most commonly seen in the ducts, which are tubes that deliver milk to the nipple, and the lacrimal gland, which is the milk-producing center. This form of malignancy has been observed in both men and women; nevertheless, women are significantly more likely to suffer from it. This is represented in the fundamental difference between the breasts of both sexes, where malignant cells are frequently identified in milk-producing centers, lobules, milk transporting canals, and ducts [3]. An anatomy image of the woman's breasts is shown in Fig. 1, along with the most preferable and sensitive area of cancer development. Breasts are the exterior structures of the female organism, so imaging tools can identify abnormalities in it. There are various imaging approaches for early breast cancer detection such as Magnetic Resonance Imaging (MRI), ultrasound, X-Ray imaging, and Computed Tomography (CT). The most widely employed method is mammography; it is also the most effective screening tool [4]. It employs X-rays to print an image of the breast to detect cancer and provide accurate results. This method is usually followed in the age group of 50 years to 70 years. Despite its efficiency, it has several limits and downsides, one of which is the risk of wrongful convictions or negatives owing to a large number of variables analyzed for evaluation. Furthermore, because of the pressure on the breast, the mammography procedure is unpleasant for females also this procedure is not recommended for dense breasts.

More pleasant and secure alternatives for breast-testing are thermal imaging. The thermal screening process is quick, easy, safe, painless, non-invasive, and inexpensive. It can identify tumor development at a preliminary phase and is also valid for dense breasts. Breast thermography uses infrared light to detect the vasculature energy emitted out from the breast's membrane [1]. This is significant since malignant tumors have greater metabolic activity than healthy tissue. Because breast tumors very seldom develop symmetrically for each breast, physicians frequently use asymmetrical assessment to analyze breast thermogram images. The rise in temperatures aids in the detection of malignant cells by analyzing the existence of unsymmetrical thermal trends in each breast's thermogram [6]. Thermography has seen a surge in popularity in recent years, particularly for the breast diagnosis process. This is owing to the allure of its low-risk strategy and the possible development in signal processing and Artificial intelligence.

The eventual objective of current research on this subject is to develop a precise and reliable tumor diagnostic that can be used as a benchmark for breast cancer examination purposes. The area of thermal images and their implications have been resurrected as a result of recent technical developments. A breast cancer diagnosis is among the most prevalent usage of thermal imaging. Thermography, on the other hand, has not yet been accepted as a common approach for this objective. Furthermore, even though mammography is hazardous to health, even then the doctors prefer it over thermography findings. If thermal imaging improves to a reasonable level, it may be presented as a suitable alternative option.

Image segmentation is a critical step in image processing that is used in areas including object recognition, pattern classification, robotics vision, diagnostic imaging, farming, and cryptography. The categorization system may fail if the segmentation result is erroneous. The segmentation approach has been used to tackle several difficulties. Based on a given thresholding value, segmentation divides an image into multiple homogenous sections or segments with comparable qualities such as texture, colour, intensity, contrasts, shape, and size. There are two types of thresholds: bilevel and multilayer. In the basic lesson, a specific threshold value is utilized to divide the image into two homogeneous portions. The latter approaches are used to divide an image into more than two sections using a histogram of pixel intensities. Because of substantial image thresholds, choosing threshold values is critical when segmenting an image. As a result, either parametric or non-parametric approaches are used to frame it as an optimization problem.

Because existing multilevel image thresholding techniques are sometimes computationally costly, meta-heuristics optimization approaches have attracted interest such as differential evolution (DE) [7, 8], ant colony optimization algorithm (ACO) [9], gravitational search algorithm (GSA) [10], Particle swarm optimization (PSO) [11, 12], bacterial foraging algorithm (BFO) [13], cuckoo search (CS) [14], grey wolf optimizer (GWO) [15], harris hawks optimization (HHO) [14], and moth-flame optimization (MFO) [16]. The number of thresholds employed in this experiment can affect the study's importance. Numerous meta-heuristic approaches used to solve various optimization issues usually have flaws such as entrapment in local regions, premature conversion, and insufficient global searchability. Scholars may now suggest improved and hybrid versions as well as improved methodologies based on these findings. In optimization problems, considering a candidate and its inverse solution at the same time can speed up converging to a globally optimum solution. Opposition-based learning (OBL) and Lévy Flight are two of the most effective methods for improving meta-heuristic algorithm search performance.

The Black Widow Optimization Algorithm (BWOA) is a recently developed population-based meta-heuristic optimization approach influenced by black widow spiders' peculiar mate selection [17]. This method mimics the distinctive behavioral traits of black widow spiders by imitating their mating behavior. This approach involves different phases known as initialization, cannibalism, mutation, and convergence. When a female wants to procreate, she sprays chemicals on selected parts of her web to lure the male black widow. Animals with insufficient fitness are eliminated from the loop at this stage, resulting in early convergence. These first spiders opted to procreate the future group in pairs. The female black widow eats the male black widow either during or after mating. She then pulls preserved sperm cells from her reproductive level of functioning and releases them into egg pouches. The spiderling emerges in the egg pouches as early as eleven days after the egg is placed. They dwell together on the parental web for several days; sibling cannibalism is detected at this time. As a result, they lift off by being propelled by the wind.

### Contribution and Motivation

The BWOA is a good alternative for solving numerical optimization benchmark problems and in engineering applications [18, 19]. However, it has some drawbacks: convergence speed, stagnation in local optima, an insufficient balance between exploitation/exploration, and low diversity. The following two stages are employed in the IBWOA version: (1) Levy flights and (2) Quasi Opposition-based learning (QOBL). Levy flights are amended to enhance the exploration capability of the basic BWOA algorithm [20]. Whereas quasi opposition-based learning is presented to improve the exploitation capacity [21]. An image-processing would be evolved as the major challenge in this arena. The major goal of this study is to provide a reliable and high-performance thermal image analysis for the diagnosis of breast cancer. The presented algorithm will enhance the balance between exploration and exploitation and also prevent stuck in the local solutions. To solve the aforementioned limitations, this paper suggests the use of a metaheuristic approach to segment thermographic images for breast cancer detection. This study intends to advance image segmentation investigation by presenting an enhanced IBWOA based on QOBL and Lévy Flight method. As per the author’s best knowledge, it's the first time that BWOA has been used for image segmentation in thermal imaging of breast cancer images. The proposed IBWOA algorithm is applied in the image segmentation process. The major contributions and objectives of this paper can be summarized as follows:

- Conduct background study and literature review of thermal breast imaging, image segmentation using various optimization techniques.
- Propose an improved BWOA using levy flight process has been proposed for solving the image segmentation problem using Otsu and Kapur’s entropy as an objective function.
- A Novel quasi opposition-based learning is presented to improve the exploitation ability and balance between exploration and exploitation.
- Performance comparison of improved BWOA is performed with existing state of art techniques such as Harris Hawks Optimization (HHO) [22], LSHADE [23], Whale Optimization Algorithm (WOA) [24], Sine Cosine Algorithm (SCA) [25], Slap Swarm Algorithm (SSA) [26], and Black Widow Optimization Algorithm(BWOA) [27] and Quantitative analysis is carried out using threshold, PSNR, SSIM, and FSIM based parameters.

Organization of paper:

The remainder of the paper is structured as follows: Sect. “ Related Works” reviews related works, while Sect. “ Materials and methods” details the materials and methods. Sect. “Proposed Methodology: Improved Black Widow Optimization algorithm (IBWOA)” outlines the proposed methodology. Sect. “Experimentation setup and Results” presents and analyzes the experimental results. Finally, Sect. “Conclusions and future work” concludes the paper and discusses future research directions.

## Related works

### Thermal imaging

In medical terms, thermal imaging is the practice of applying the heat energy fluctuations released by the body parts and translating them into images that can be evaluated by professionals. This particular concept has a long and illustrious history, traced to ancient cultures. The ancient People used their symbols to sense heat generated from limbs in evaluating and treating sickness. Furthermore, Greeks used clay or mud to record the temperature of human organs, with the irregularity being discovered by watching the region which dries out first. Thermal imaging of the breasts refers to the variation in the heat map deep within the skin among normal and cancerous tissues. The presence of a tumor in the body raises the heat of the cells and around it [28]. A balanced assessment of normal and cancerous tissues is generally used by professionals. The process for utilizing thermography to screen for breast cancer is relatively simple. It begins with a visual examination of the area of the chest. This enables doctors to link any unexpected activity to the heat map.

### Image segmentation

For diagnosing breast cancer, image segmentation is a necessary stage. The classification system may fail if the segmented outcome is incorrect. To make the analytical stage easier, segmentation divides an image into various parts based on recognized information such as color, pattern, intensity, or movement [29, 30]. A visualization, detection, identification, and quantifying assessment are usually performed after a segmentation technique. Furthermore, thresholding has been widely used in the automated process of medical image analysis as a means of assisting doctors in the diagnosis phase. Even though there are numerous works on fully automated and semi- automated segmentation, the assessment of images and evaluation remains a challenge even today also. The major reason behind this is the complex frameworks with common characteristics, noise situations, poor contrast, and inferior boundaries, all of which are common in medical images. Support Vector Machine (SVM) [31–33], decision tree, K-nearest neighbor (k-NN) [26, 34, 35], Bayesian network (BN) [36], artificial neural network (ANN) [35, 37, 38], deep learning (DL) [39–41], and convolutional neural networks (CNNs) [42–44] are widely used method in machine learning for classification and problem analysis.

The early thermography trials for breast cancer detection were ineffective due to thermal imaging technology's inability to monitor temperature differences. Cameras have grown more sensitive as a result of technological advancements, and temperature disparities in breast infrared thermography of tumor patients have been emphasized.

Thermal imaging are useful for breast cancer diagnosis, assessment of benign diseases, and follow-up operations since 2014 [32, 42]. In this context, as explained, thermal imaging has become the focus of various investigations involving breast cancer diagnosis in recent years. We'll go through a few of these in this subsection. The development of a Computer-Aided Diagnostic (CAD) approach for breast cancer classification always begins with the segmentation of a Region of Interest. The goal of Region of Interest segmentation is to isolate the breast areas from the surrounding tissue. The researchers of [3] presented a thermography-based breast cancer classification approach as a novel process for classification. This approach is based on categorizing breast thermal images into three groups: healthy, harmless, and cancerous. Pre-processing stage and segmentation, extraction of features, feature selection employing ant colony optimization and particle swarm optimization, and classification using a Multi-class support vector machine are the primary phases in this approach. The integration of the curve variable k and the gradient vector flow is employed as a segmentation approach [45]. To characterize the segmented breast cancer dataset, the authors used a convolutional neural network (CNN). They employed a mix of binary masks, k-means clustering, and the signature border for feature extraction. In the study [46] AlFayez et al. utilized the Multilayer Perceptron (MLP) and Extreme Learning Machine (ELM) as classification techniques (ELM). Ibrahim et al. [47] suggested a horizontal projection profile (HPP) examination to segment both the right and left breasts by locating the top, left, lower, and right boundaries. HPP was utilized to identify the top and bottom boundaries, while Vertical Projection Profile (VPP) was utilized to identify the left and right borders. After employing HPP, Sathish et al. [48] applied asymmetric assessment, a novel alternative for segmentation that relies on locating the point of intersection to separate the right and left breasts [28, 48].

Shahari S and Wakankar [49], utilized the segmentation technology known as hot region segmentation strategy, which relies on explicitly dividing objects from backdrops after implementing the k-means clustering algorithm, that was implemented to categorize colors for Lab mode after converting from RGB mode to show and compare the distance between colors. Gonçalves et al. [33] relied on using three kinds of thermal images for an individual, whether healthful, normal, or dangerous, to diagnose breast cancer using machine learning algorithms. They used a mix of feature extraction algorithms following segmenting the ROI. Hossam et al. introduced a novel automated segmentation approach in [38], which included pretreatment, segmentation, and segregation for an area of interest, followed by ROI segmentation towards the image. Just the segmentation ROI was subjected to the feature extraction technique. Ultimately, support vector machines (SVM) and artificial neural networks were used to generate output. Multilevel thresholding of Breast Thermal images using the Dragonfly algorithm was proposed by Díaz-Cortés et al. [1]. The temperature distribution of the photos is used as a source for segmenting grayscale breast infrared images. [50] suggest colour segmentation of aerial photos using nature-inspired optimization techniques. As objective functions, Otsu's between-class variance and Kapur's entropy were used to evaluate the techniques' effectiveness. For colour segmentation of satellite images, Kapur's entropy-based objective function performs better, while the Cuckoo's search strategy is more economical. He and Huang [51] proposed an effective krill herd (EKH) optimization strategy for multilayer thresholding of colour images, based on Otsu's method with Kapur and Tsallis entropy as objective functions. When compared to the krill herd algorithm, the effective krill herd (EKH) method performs better krill herd (KH). Oliva et al. [52] offer a multilayer thresholding approach based on the electromagnetism optimization (EMO) algorithm. The objective functions are Otsu's and Kapur's entropy criteria, and the source is a histogram of photographs. For image segmentation, Samantaray et al. [14] proposed a hybrid Artificial Bee colony-Salp Swarm algorithm (ABC-SSA). In a multilevel thresholding issue utilizing Kapur's entropy as the objective function, the hybrids technique outperforms the ABC [53], Sine Cosine Algorithm (SCA) [25], Social Spider Optimization (SSO) [54], and SSA algorithms [26].

Pare et al. [55] presented multilevel thresholding of satellite images utilizing optimization algorithms Wind Driven Optimization (WDO) [56], Bacterial Foraging Optimization (BFO) [57], Firefly Algorithm (FA), Artificial bee colony algorithm (ABC), Differential evolution (DE), and Particle swarm optimization (PSO) with image energy curves as input. Kapur's entropy, Tsallis entropy, and Otsu's approach are utilized as objective functions. The entropy-based DE algorithms developed by Kapur generate superior segmented images. Bhandari et al. [56] presented multilevel thresholding utilizing WDO and CS, utilizing Kapur's entropy as the aim function. The studies describe multilevel thresholding employing multiple optimization strategies. Using maximizing Otsu's, Kapur's, and Tsallis's entropy, Bhandari et al. [58] suggested a modified artificial Bee colony (MABC) optimizer for multilayer thresholding of aerial photos. When compared to the ABC approach, the MABC technique provides superior segmented images. Acharya et al. [59] uses a support vector machine (SVM) for 50 breast images for automatic diagnosis and classification using the texture feature. Milosevic et al. [34] presented classification, and segmentation of breast thermal images using SVM, Naive Bayes classifier, K Nearest Neighbor classifier, and GLCM features.

Nonetheless, these methods have drawbacks; for instance, they are computationally costly, especially as the number of thresholds grows [12]. As a result, multilevel thresholding is seen as a unique issue that must be overcome. Meta-heuristic approaches are extensively used in the associated literature to tackle these difficulties on these grounds. Nature inspires metaheuristic algorithms to find application in many areas like physics, biology, and social behavior, among other subjects. Many researchers have utilized them to discover the ideal values for real-world situations because of their ease of implementation, versatility, and good performance. Many meta-heuristic algorithms have been presented in recent years like swarm intelligence (SI) [60] like Particle Swarm Optimization (PSO) [11], Ant Colony Optimization (ACO) [61], Artificial Bee Colony (ABC) [53], Teaching Learning Based Optimization (TLBO) [62], Gray Wolf Optimization (GWO) [63], Salp Swarm Algorithm (SSA) [26], and evolutionary computing (EC) [64] like Differential evolution (DE) [65], etc. Along with all these optimizations many different modified versions of these algorithms have also been proposed for example Cuckoo Search Algorithm via lévy flights, Learning enthusiasm-based TLBO (LebTLBO) [66, 67], Modified Naked Mole Rat Optimization (mNMRO) [68], etc. Zhao et al. proposed a variant of the Slime Mould Algorithm (SMA) [69], which had been used for the segmentation of computed tomography (CT) images using multilevel thresholding using Renyi’s entropy as the primary objective function. Many such algorithms have been presented recently to improve the superiority of image segmentation.

## Materials and methods

To understand our work, this part gives the thoughts about the materials and procedures necessary for the construction of the suggested segmentation approach.

### Dataset

**Fig. 2:**
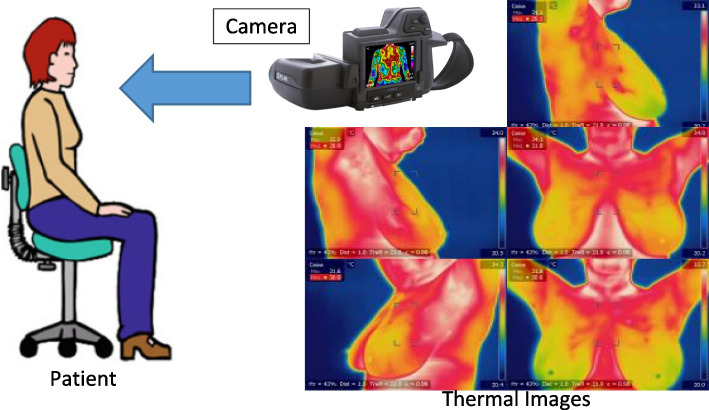
Thermal imaging mechanism (Image Dataset [Online]. Available: http://visual.ic.uff.br/dmi/prontuario/home.php)

The person must then normalize by being at ambient temperature (18 °C to 25 °C) for fifteen minutes. The person must strip down the upper half of his or her body, above the waistline to the neck. The whole mechanism and arrangement are well explained in Fig. 2. In which the patient at room temperature is exposed in front of an IR camera and corresponding thermal images at different positions are shown. The dataset for breast cancer is taken from Digital Database for Screening Mammography (DDSM): Breast Cancer Image Dataset [70] (http://visual.ic.uff.br/dmi/prontuario/home.php). Breast cancer may be detected via thermal imaging analysis, which involves multiple processes like preprocessing, segmentation, and classification of features. Thermal image processing' preprocessing and segmentation phases are believed to be the most important procedures in identifying cancerous tissue since they may enhance the precision of retrieving information and the classification of normal and abnormal situations. The contour (breast area) may be retrieved from the thermal image in the preliminary step by deleting undesirable parts such as the neck and shoulders. This is accomplished by transforming photos to grayscale for segmentation. The three phases of preprocessing include identifying the region of interest, improving the thermal image, and normalizing the image matrix.

### Methods

To perform segmentation based on image thresholding, Otsu’s between-class variance and other maximum entropy methods like Kapur’s entropy [71], Renyi’s entropy [72], and Tsallis entropy [61] have been developed. This kind of approach combines information theory successfully, but the probability of a gray level value being shown primarily affects the methodologies. Another reason for influencing or affecting the segmentation results is to ignore the gray level value of the pixels. In 1979, Otsu proposed a thresholding method that maximizes between-class variance and minimizes intraclass variance to achieve optimum threshold values [73]. The brightness and contrast of an image do not affect the 1-D Otsu method, furthermore, it is a procedure with the less computational cost for a small number of thresholds. The segmentation results of an image using Otsu are better. Nevertheless, the algorithm primarily considers only the gray level value, for that reason, it fails to produce optimum results in the case of noisy images. Depending upon the number of threshold values, thresholding may be defined by bi-level (BT) or multi-level (MT) thresholding. Bi-level thresholding divides an image into two distinct regions, while MT creates multiple regions in an image.

#### Otsu between-class variance

The between-class variance introduced by the Otsu is a nonparametric automatic method for image segmentation. It employs techniques to determine a threshold value to separate a histogram into different groups [74]. Conventional techniques offer bi-level thresholding image thresholding but due to the tiny differences between the object and the context of a complex image, bi-level thresholding is unable to properly determine the ideal threshold. Hence, the segmentation problem has been reported as a multilevel problem in literature, but practically it becomes difficult to implement. Otsu's and Kapur's approaches are therefore inappropriate for multilevel image segmentation applications in the real world. According to the literature, 3–4 threshold values will cover all the points in the histogram, thus there is no need to go beyond that [53, 75]. An exhaustive search for multilevel segments will result in an exponential increase in processing time as the number of thresholds rises. When it comes to the usage of photographs, it might be tough to know where to start. Discern the valleys and bottoms, particularly in circumstances when the valley is wide and flat, with a lot of noise. The data in this incident concerning surrounding pixels in the original image might change to make the histogram better helpful for thresholding. This approach is straightforward and unaffected by the image's intensity or brightness. The classic Otsu approach, on the other hand, is a solitary classification technique. The object inside the two classes could no longer be differentiated after applying the method to split the image into two classes. Other targets can be distinguished between the two classes; consequently, these two classes must be further divided. Different classes are segmented using the multi-threshold Otsu approach. To split the actual photo into *d* classes, *d-1* thresholds are required for the multi-level technique. As a result, the array of thresholds used for image segmentation is represented as th = [th_1_, th_2_, th_n_,..., th_d-1_]. Each class may be defined in Eq. 1.

1 $$\begin{array}{c}{C}_{1}\leftarrow \left\{0, 1, \dots ., {th}_{1}\right\}\\ {C}_{n}\leftarrow \left\{{th}_{n}+1, {th}_{n}+2, \dots ., {th}_{n+1}\right\}\\ .\\ .\\ {C}_{d}\leftarrow \left\{{th}_{d}+1, {th}_{d}+2, \dots ., L-1\right\}\end{array}$$

The interclass variance/ between class variance ${\sigma }_{B}^{2}$ is given by the following equation.

2 $${\sigma }_{B}^{2} ={\omega }_{1}{\left({\mu }_{1}-{\mu }_{T}\right)}^{2}+{\dots \dots .\omega }_{n}{\left({ \mu }_{n}-{\mu }_{T}\right)}^{2}+{\dots \dots .\omega }_{d}{\left({ \mu }_{d-1}-{\mu }_{T}\right)}^{2}$$

Where ${\omega }_{0} \text{and} {\omega }_{n}\text{ are}$ the probability of the same greyscale pixel of classes ${C}_{1}$ and ${C}_{n}$ given in Eq. 1, ${\mu }_{0}$ and ${\mu }_{n}$ for average pixel levels of classes ${C}_{1}$ and ${C}_{n}$. The expression of ${\mu }_{1}$ and ${\mu }_{n}$ are given by Eq. 3.

3 $${\mu }_{1 }= \frac{\sum_{i=0}^{n}{ip}_{i}, }{{\omega }_{1}}, { \mu }_{n}= \frac{\sum_{i={th}_{n}+1}^{{th}_{n+1}}{ip}_{i} }{{\omega }_{n}} ,\dots \dots { \mu }_{d}= \frac{\sum_{i={th}_{d-1}+1}^{L-1}{ip}_{i} }{{\omega }_{d-1}}$$

4 $${\omega }_{a}= \sum_{i=0}^{n}{p}_{i}, , {\omega }_{b}= \sum_{i={th}_{n}+1}^{{th}_{n+1}}{p}_{i} ,\dots \dots { \omega }_{d}=\sum_{i={th}_{d-1}+1}^{L-1}{p}_{i}$$

It is necessary to compute the probability distribution ${p}_{i}$ that is given by Eq. 5

5 $${p}_{i}=\frac{{n}_{i}}{N}$$

where ${n}_{i}$ is the number of pixels having grey level *i* and *N* is the total number of pixels. In Otsu’s method, the intraclass variance is calculated and it provides optimum threshold values. Considering *i* as a certain class for a given image $b$ having *L* gray levels (1,2,…, *L*) in the range [0, *L*-1]. Extended between the class value is given by $f\left(k\right)$ between-class variance is represented in Eq. 6.

6 $$f\left(k\right)=\sum_{i=1}^{M}{\omega }_{i}{\left({\mu }_{i}-{\mu }_{T}\right)}^{2}$$

while considering the above classes Otsu method can be easily extended to multilevel thresholding for *M-1* thresholding levels. Where ${\omega }_{i}\text{ is a}$ zeroth-order cumulative moment for *i*^*th*^ class and ${\mu }_{T}$ is mean intensity for hole image.

7 $${f}_{OTSU}(T)={\varnothing }_{o}=\text{Arg max}\left(f\left(k\right)\right), 0\le k\le L-1$$

where ${f}_{OTSU}$ is an objective, and the required optimal threshold value of pixel can be derived from it by maximizing Eq. 7. Fitness function considering *i* multilevel threshold values is given by the following equation.

8 $${f}_{OTSU}({T}_{i})={\varnothing }_{o}=\text{Arg max}\left(f\left({k}_{i}\right)\right), 0\le k\le L-1, i=1, 2, \dots .,\text{ d}-1$$

#### Kapur Entropy

Kapur's entropy approach has caught the considerable interest of researchers and is commonly utilized for image segmentation problems due to its amazing results. For multilevel thresholding segmentation, Kapur's entropy is a useful and practical statistic. The image is divided into separate classes using Kapur's entropy, and the amount of the entropy decides if the group is homogeneous. Based on information theory, Kapur's entropy methodology shows the ideal thresholding values by maximizing the entropy of every separate class or the summation of entropies. Kapur's entropy has a simple mathematical and very simple procedure to be followed and provides a significant level of stability. It possesses many positive points such as fast processing, a high level of classification performance, and also provides viable separation between distinct classes based on the entropy of the original image. By maximizing the objective function value, Kapur's entropy discovers the optimal threshold values. To tackle the problem of image segmentation different automatic processes are evolved, which can choose the optimum statistical characteristic and threshold automatically. The value of entropy ‘$H$’according to the Shannon theorem is given by Eq. 9.

9 $$H=-\sum_{i=1}^{n}{P}_{i}{log}_{2}{P}_{i}$$

Where with ${P}_{i}$ as the possibility of the *i*^th^ gray level and ‘n’ defines the total grey level number. Kapur’s entropy is used to find a single optimal threshold value by maximizing the below expression.

10 $${f}_{Kapur}(T)={H}_{A}+{H}_{B}$$

Different entropies associated with distinct classes may be described as the following equation

11 $${H}_{A}=-\sum_{i=0}^{{t}_{1}-1}\frac{{p}_{i}}{{\omega }_{A}}ln\frac{{p}_{i}}{{\omega }_{A}}, {\omega }_{A}= \sum_{i=1}^{{t}_{1}-1}{p}_{i}$$

12 $${H}_{B}=-\sum_{i={t}_{1}}^{L-1}\frac{{p}_{i}}{{\omega }_{B}}ln\frac{{p}_{i}}{{\omega }_{B}}, {\omega }_{B}= \sum_{i={t}_{1}}^{L-1}{p}_{i}$$

In order to extend the Kapur’s entropy from bi-level to multilevel thresholding further entropy classes may be added as given in Eq. 13.

13 $$\begin{array}{c}{H}_{A}=-\sum\limits_{i=0}^{{t}_{1}-1}\frac{{p}_{i}}{{\omega }_{A}}ln\frac{{p}_{i}}{{\omega }_{A}}, {\omega }_{A}= \sum\limits_{i=1}^{{t}_{1}-1}{p}_{i} \\ {H}_{B}=-\sum\limits_{i={t}_{1}}^{{t}_{2}-1}\frac{{p}_{i}}{{\omega }_{B}}ln\frac{{p}_{i}}{{\omega }_{B}}, {\omega }_{B}= \sum\limits_{i={t}_{1}}^{{t}_{2}-1}{p}_{i}\\ {H}_{c}=-\sum\limits_{i=0}^{{t}_{3}-1}\frac{{p}_{i}}{{\omega }_{C}}ln\frac{{p}_{i}}{{\omega }_{C}}, {\omega }_{C}= \sum\limits_{i=1}^{{t}_{3}-1}{p}_{i} \\ {H}_{m}=-\sum\limits_{i={t}_{m}}^{L-1}\frac{{p}_{i}}{{\omega }_{m}}ln\frac{{p}_{i}}{{\omega }_{m}}, {\omega }_{m}= \sum\limits_{i={t}_{1}}^{L-1}{p}_{i}\end{array}$$

Kapur's entropy is used as an objective function to find optimal thresholding values by maximizing the following function given by Eq. 14 [76].

14 $${f}_{Kapur}(T)={\varnothing }_{k}=\text{arg max}\sum_{i=0}^{m}{H}_{i}(th), 0\le th\le L-1$$

${f}_{Kapur}\left(T\right),$ will provide multiple optimal threshold values as mentioned in Eq. 15 [56]

15 $${f}_{Kapur}\left(T\right)={f}_{Kapur}\left({th}_{i}\right), i=\text{1,2},3,\dots \dots \dots \dots k$$

where $T$ represents a vector having multiple threshold values ${th}_{1},{th}_{2},{th}_{3},\dots ..{th}_{k-1}$ and $\text{i}$ correspond to a specific class.

### Black Widow Algorithm

**Fig. 3:**
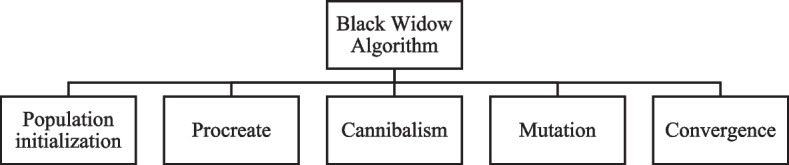
Different phases of the original BWOA algorithm

The Black Widow Optimization (BWOA) is a recently developed population-based meta-heuristic optimization approach for solving complicated engineering optimization issues [27]. This method mimics the distinctive behavioral traits of black widow spiders by imitating their mating behavior. The black widow spider belongs to the Araneae family and has eight legs. The infamous and well-known black widow spiders belong to the Latrodectus subfamily of spiders. Latrodectus is a genus of spiders that includes the black widow. The black widow weaves her web at all times of the day and night, while the female widow spends most of her adult life in a similar location. When a female black widow wants to mate, she marks a few locations on her web to attract the male. The suggested technique, like other conventional methods, starts with an initializing spider population, with each spider representing a potential value. The spiders' initializations are in couples and are attempting to generate new offspring. A female spider consumes the male subsequent after or during or post pairing and eventually transports the sperm to the egg sacs. After one and a half weeks of placement, offspring emerges from the egg vesicles. The offspring will stay in the mother web for many weeks and during this stay time sibling cannibalism is decided. As a result, the spiderlings are blown away from the web. The next section outlines the entire procedure of the BWOA methodology phases. It has four phases as described in Fig. 3.

#### Population initialization

The variable values of the problem parameter must create an adequate framework for the resolution of the present issue to solve an optimization problem. This framework is referred to as a “chromosome” in GA and a “particle position” in PSO, but it is referred to as a “widow” in the BWOA. The possible answer to each challenge has been modeled like a Black widow spider in the BWOA. The findings of the issue variables are shown on each Black widow spider. The architecture should be treated as an array in this study can fix objective functions. The black widow spider evaluates every problem's possible solution. Each black widow spider displays the values of the issue variables. The solution provided by BWOA is referred to as *widow* given by Eq. 16 and it generates an array of 1 × *K*_var_ for the problem of dimension *K*_var._

16 $$widow=\left[{x}_{1}, {x}_{2},\dots \dots \dots .{x}_{{K}_{var}}\right]$$

Every value of the array $\left[{x}_{1}, {x}_{2},\dots \dots \dots .{x}_{{K}_{var}}\right]$ will be a floating-point number. The fitness of a widow is determined by Eq. 17 applying the fitness model *F* to *widow* of $\left[{x}_{1}, {x}_{2},\dots \dots \dots .{x}_{{K}_{var}}\right].$

17 $$F(widow)=F\left({x}_{1}, {x}_{2},\dots \dots \dots .{x}_{{K}_{var}}\right)$$

The optimization method is started by populating the spider's population with a prospective widow matrix of size $${K}_{var},$$$${K}_{pop}$$. Then, through mating, a pair of parents is chosen at random to carry out the procreating stage, in which the male black widow is consumed by the female black widow during or after mating.

#### Procreate

Because the pairings are self-governing, they start to mate to replicate the next population. As a result, each pair spontaneously mates in their web, regardless of the possibilities. In the physical realm, each pairing produces over a million eggs; nonetheless, Finally, some of the web infants that are muscular are saved. This is where we are right now with this strategy. In the real world, each mating can produce approximately 1000 eggs, but only the strongest spiderlings survive. Similarly, in this algorithm, to facilitate reproduction, an matrix called alpha ($\alpha )$ is created along with a widow array containing random numbers. After that, progeny is formed by exploitation the array of random numbers. In this context, ${x}_{1}$ and ${x}_{2}$ represent father and mother, while ${V}_{1}$ and ${V}_{2}$ represent the children, as described in Eq. 18.

18 $$\left\{\begin{array}{c}{V}_{1}=\alpha \times {x}_{1}+(1-\alpha )\times {x}_{2}\\ {V}_{2}=\alpha \times {x}_{2}+(1-\alpha )\times {x}_{1}\end{array}\right\}$$

#### Cannibalism

**Fig. 4:**
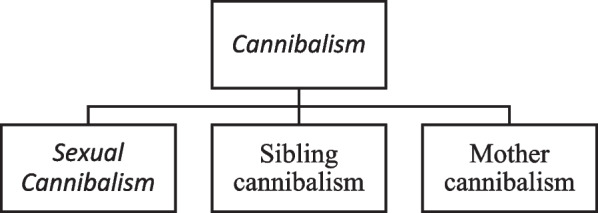
Different phases of Cannibalism

In this phase, three different Cannibalism processes will happen and they are given in Fig. 4. First is sexual cannibalism, in which a female widow will eat a male widow. The gender of the widow will be identified by their respective fitness function value.

The second type is sibling cannibalism, in this only fittest will survive and the strong candidate will eat the weak sibling. The number of survived offspring will be determined by the cannibalism rating denoted by CR. In a few cases, even mother widow is eaten by their baby spiders.

#### Mutation

**Fig. 5:**
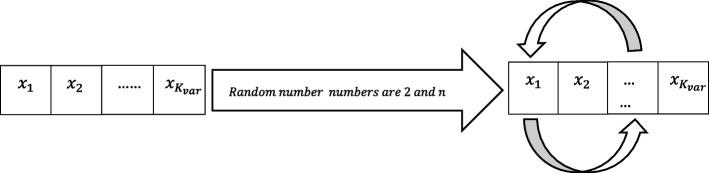
Mutation process

The mute pop quantity is randomly determined within the population during the mutation process. Every response can shift two components in the array at the random structure as mentioned in Fig. 5. The mutation rate is calculated using *Mute*_*pop*_ data.

#### Convergence

There will be three stopping/ termination conditions that can be tested in three phases, similar to previous algorithms: (1) obtaining the stated degree of accuracy, (2) observing no fluctuation in the fitness value, and (3) obtaining the required degree of precision. The BWOA is used to solve several benchmark optimization issues, and the best solutions are gathered.

## Proposed methodology: Improved Black Widow Optimization algorithm (IBWOA)

The Black Widow Optimization (BWOA) is a population-based meta-heuristic optimization technique for tackling difficult engineering optimization problems that were recently created [27]. This approach imitates the mating activity of black widow spiders to emulate their particular behavioral features. The black widow spider has eight legs and is a member of the Araneae family. The Latrodectus subfamily of spiders includes the famed and well-known black widow spiders. The black widow spider belongs to the Latrodectus genus of spiders. The female widow lives most of her adult years in a similar position as the black widow, who builds her web at all hours of the day and night.

### System model

Improved Black Widow Optimization algorithm (IBWOA) will include two major modifications to its existing basic structure. Lévy flight process is used to drive the procreation stage, and hence it will enhance the exploration capability of the basic Black Widow Optimization algorithm and enrich the exploitation capacity and create a balance between the exploration and exploitation quasi opposition-based learning (QOBL) is adopted. Further, details of Lévy flights, opposition-based learning (OBL), and quasi-opposition-based learning (QOBL) are provided in the following subsections.

#### Lévy flights

By construction, Lévy flights are Markov processes and are a representation of a random process [20], which involves step length as Lévy distribution. To minimize algorithmic stagnation and entrapment in local minima, the Lévy flight distribution is included in the BWOA approach. It also helps to improve the exploring power and exploration potential of this optimization method by boosting its chance of creating novel solutions. Lévy flight is a random procedure for producing a new response based on an arbitrary walk with Lévy steps. The following is the new population position based on the Lévy distribution [69]. This step will model the IBWOA and update the matrix alpha with ${\alpha }{\prime}$. Also, new offspring will be named as ${V}_{1}^{new}$ and ${V}_{2}^{new}$ calculated according to Eq. 19

19 $$\left\{\begin{array}{c}{V}_{1}^{new}={\alpha }{\prime} \times {x}_{1}+(1-{\alpha }{\prime} )\times {x}_{2}\\ {V}_{2}^{new}={\alpha }{\prime} \times {x}_{2}+(1-{\alpha }{\prime} )\times {x}_{1}\end{array}\right\}$$

The Lévy flight is characterized by the combination of small and sporadically large step sizes, which enhances the search capability of the model during optimization. The standard Lévy flight distribution parameter for step size $(L\acute{e} vy)$ is given by Eq. 20.

20 $$L\acute{e} vy=0.01 \times \frac{{R}_{6}}{{\left|{R}_{7}\right|}^{ \frac{1}{\beta }}}$$

where, ${R}_{6}$ and ${R}_{7}$ are normal distribution arbitrary values consist of the standard deviation ${\sigma }_{{R}_{6}} and {\sigma }_{{R}_{7}}$ respectively, and are calculated as ${R}_{6}=normal\left(0, {\sigma }_{{R}_{6}}^{2}\right) \text{and} {R}_{7}=normal(0, {\sigma }_{{R}_{7}}^{2})$. The Lévy flight ${\sigma }_{{R}_{6}}$ is formulated by Eq. 21.

21 $${\sigma }_{{R}_{6}}={\left(\frac{\Gamma (1+\beta )\times \text{sin}\left( \frac{\pi \beta }{2}\right)}{\Gamma \left(\frac{1+\beta }{2}\right)\times \beta \times 2\left( \frac{\beta -1}{2}\right)}\right)}^{\frac{1}{\beta }}$$

where $\Gamma$ corresponds to standard gamma function, and β is in the limit [1, 2] and assumed to be 1.5 [77].

#### Opposition-based learning

Tizhoosh suggested opposition-based learning (OBL) [78] as an effective search means to prevent convergence speed in 2005. The central concept behind OBL is to produce the opposite result in the search area given by Eq. 22 and then use the objective function to assess both the initial and opposing solutions. The best option will then be kept and used in the following iteration. Generally, the OBL technique has a higher chance of providing optimum answers that are closer to each other than random solutions.

22 $${y}_{obl, i}\left(t\right)=u+l-{x}_{i}\left(t\right) , i \in [1, 2,\dots \dots \dots .., n]$$

Where, ${x}_{obl, i}\left(t\right)$ is the opposite solution presented by the current given solution ${x}_{i}\left(t\right)$ at given time *t*.

#### Quasi Opposition-Based Learning

Rahnamayan et al. [21] presented a variation of OBL dubbed quasi-opposition-based learning (QOBL) depending on the given description. The QOBL technique, unlike OBL, used a quasi-opposite result instead of the opposing solution. As a result, the QOBL technique outperforms the prior strategy in terms of discovering globally optimum solutions. The quasi-opposite response may be computed using the fundamental principle of opposing solutions by Eq. 23:

23 $${x}_{qobl, i}\left(t\right)=\text{rand}\left( \frac{u+l}{2}, {x}_{obl, i}\left(t\right)\right)$$

**Fig. 6:**
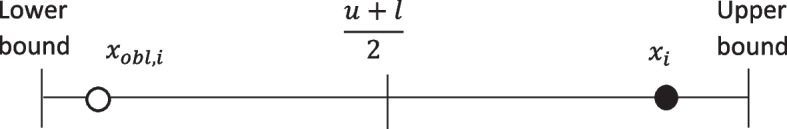
Representation of Quasi Opposition-Based Learning

Where, ${x}_{obl, i}\left(t\right)$ is the opposite solution, ${x}_{qobl, i}\left(t\right)$ is quasi opposition-based learning solution, $u$ is upper bound, and $l$ is lower bound. The pictorial representation and scheme of quasi-opposition-based learning (QOBL) are shown in Fig. 6.

The proposed IBWOA algorithm enhances the existing BWOA by combining the Lévy flight process and quasi-opposition-based learning with the standard BWOA algorithm. The pseudo-code of the IBWOA is explained in Algorithm 1.

**Figure:**
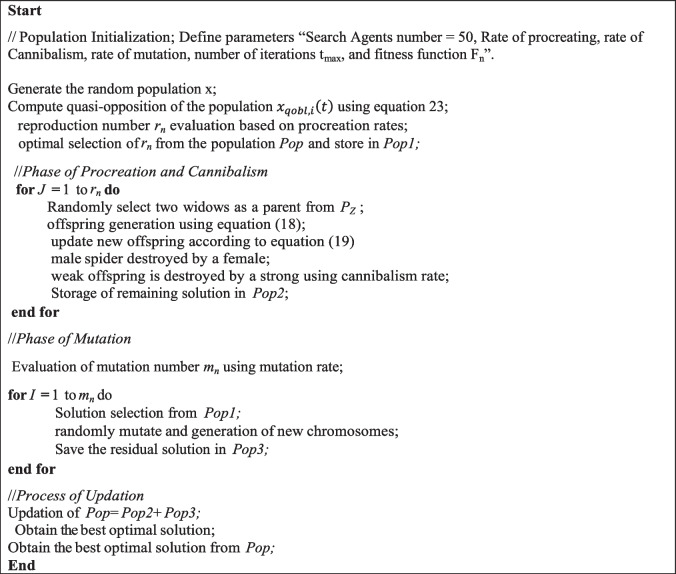
** Algorithm 1.** Improved Black Widow Optimization Algorithm (IBWOA)

## Experimentation setup and results

IBWOA is programmed in Matlab2021, implemented on windows 10 over a 64-bit environment on a computer with an Intel Core I7 processor, and 10 GB memory. The parametric value chosen for different variables is chosen as a percent of Crossover 0.8, 51 independent runs, the maximum number of 350 iterations for each test image, mutation value 0.4, cannibalism 0.5, and size of population taken as 50. For comparative analysis six meta-heuristic algorithms are considered, which are Harris Hawks Optimization (HHO) [22], LSHADE [23], Whale Optimization Algorithm (WOA) [24], Sine Cosine Algorithm (SCA) [25], Slap Swarm Algorithm (SSA) [26], and Black Widow Optimization Algorithm(BWOA) [27].

### Experimental setup

**Fig. 7:**
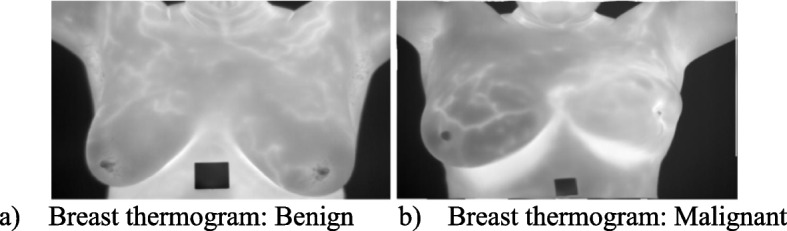
Representation of benign and malignant cases. **a** Breast thermogram: Benign. **b** Breast thermogram: Malignant

All the parameters in the experiment are fixed as per the default values proposed in the basic research paper. The method was evaluated across 35 independent runs with a maximum of 350 iterations for each test image for an unbiased test image comparability. For a valid comparison, all methods use the same simulation environment. The application of the IBWOA for thermal image segmentation is described in this study. This approach is tested specifically in the instance of Breast Thermography. To qualitatively and quantitatively examine the data, a group of 10 photos from the database is randomly picked from the complete database of digital database for Screening mammography (DDSM): breast cancer image dataset [70]. Artificial objects such as tags, gauges, and branding are removed from the photos to emphasize segmentation. The random images having positive and negative cases are considered for experimentation and Fig. 7 represents a positive and a negative case image. The optimum values are shown in bold values. These ten test images and their corresponding histogram graphs are represented in the Table 1.

**Table 1:**
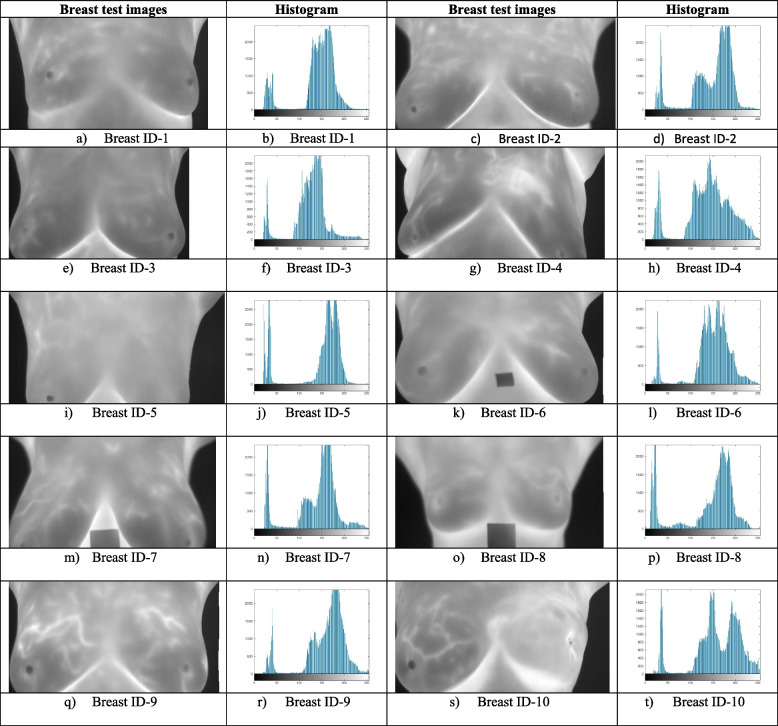
Dataset along with associated histogram

### Experimental parameters

The peak signal-to-noise ratio (PSNR) indicates the amount of noise present in the resultant image as compared to the original image [79–81]. The PSNR between original or ground truth ${I}_{G}$ and the segmented image ${I}_{th}$ is calculated as mentioned in Eq. 24 and the RMSE is provided by Eq. 25.

24 $$PSNR=20\, {log}_{10}\frac{255}{RMSE} (dB)$$

where

25 $$\text{RMSE}=\sqrt{\frac{\sum_{i=1}^{M}\sum_{j=1}^{N}\left({I}_{G}-{I}_{th}\right)}{M X N}}$$

where the size of an image is M × N, a higher PSNR value is desirable and it represents less amount of noise that has been added during the processing [82]. Structural Similarity Index is another parameter used to evaluate given by the following Eq.

26 $$SSIM\left(I,{I}_{s}\right)=\frac{(2{\mu }_{I}{\mu }_{{I}_{S}}+{C}_{1})(2{\sigma }_{I, { I}_{S}}+{C}_{2})}{({\mu }_{I}^{2}+{\mu }_{{I}_{S}}^{2}+{C}_{1})({\sigma }_{I}^{2}+{\sigma }_{{I}_{S}}^{2}+{C}_{2})}$$

where ${\mu }_{{I}_{S}}({\sigma }_{I, { I}_{S}})$ and ${\mu }_{I} \left({\sigma }_{I}\right)$ are the mean intensity of images IS and I, respectively, where ${\sigma }_{I, { I}_{S}}$ is the governance of I and IS, and ${C}_{1}$ and ${C}_{2}$ coefficient values are equal to 6.5025 and 58.52252, respectively.

The Feature Similarity Index (FSIM) is an important parameter used to estimate the similarity between the original image and the segmented image. Its value lies between the range of [-1, 1] and a higher value is desired and given by the following equations.

27 $$FSIM=\frac{\sum {S}_{L}{PC}_{m}}{\sum {PC}_{m}}$$

28 $${S}_{L}={S}_{PC}\times {S}_{G}$$

29 $${S}_{pc}=\frac{{2PC}_{1}{PC}_{2}+{T}_{1}}{{PC}_{1}^{2}+{PC}_{2}^{2}+{T}_{1}}$$

30 $${S}_{G}=\frac{{2G}_{1}{G}_{2}+{T}_{2}}{{G}_{1}^{2}+{G}_{2}^{2}+{T}_{2}}$$

Where ${PC}_{1}$ is phase consistency of the original image, ${PC}_{2}$ phase consistency of segmented image and ${T}_{1}$ is a positive constant. Where ${G}_{1}$ & ${G}_{2}$ are the gradient constant of the original and segmented image respectively and ${T}_{2}$ is again a constant positive.

A comparative analysis is carried out with existing state-of-the-art multilevel thresholding techniques like Harris hawks optimization (HHO) [22], LSHADE [23], Whale optimization algorithm (WOA) [24], Sine cosine algorithm (SCA) [27], and Black widow optimization [27]. The parameters selected for comparison with the above-mentioned techniques are threshold values, Peak signal-to-noise ratio (PSNR) [74], Structural similarity index module (SSIM) [83], and Feature similarity index (FSIM).

#### Result and discussion: IBWOA based on Otsu’s results

This subsection provides the results based on Otsu’s fitness function as given in Eq. 8 using IBWOA. The suggested IBWOA-based breast thermal image segmentation approach is compared to different algorithms using Otsu's method to obtain the best potential threshold value, applied over ten thermography images. The threshold values for 2, 3, 4, and 5 levels are depicted in Table 2. It is noticeable that most algorithms give the same threshold values for the 2nd and 3rd levels but show different values for higher thresholding levels. The computational solution of the suggested technique, the IBWOA algorithm, is presented and discussed in this subsection. Equation 8 uses Otsu's between-class variance as a fitness function. For all the thermal imaging test images utilized in the studies, Tables 4 and 5 show the segmented images derived from the proposed IBWOA with various levels of thresholds, including [Th = 2, 3, 4, 5]. The distribution of the best-selected thresholding values over the histogram is also included in the findings. Table 3 presents the results after applying the IBWOA-Otsu method to the thermography images. It has three sub-parts: the first part shows segmented images after deploying Otsu, the second part demonstrates the histogram graph plotted with the best/optimum histogram values, and the third part provides convergence graphs. Convergence graphs depict how many iterations are required for the thresholding results to stabilize. It's critical to maintain track of computation convergence. Convergence graphs show how a method improves over time, which is important for determining its effectiveness. Table 3 shows superior convergence results. It is also concluded from the convergence results that the computational complexity is not very high, as IBWOA achieves optimum results even before 10 iterations for two, three, and four threshold values. Segmentation using five threshold values is a little more complex, as it takes more iterations to reach optimum values.

**Table 2:** The Otsu’s optimal thresholds values

Test Image	Th	HHO	LSHADE	WOA	SCA	SSA	BWOA	IBWOA
Test-image1	2	87 155	87 155	87 155	87 155	87 155	87 155	87 155
	3	85 149 175	85 149 175	85 149 175	83 148 176	85 149 175	85 149 177	85 148 174
	4	83 141 161 184	83 141 161 184	83 141 161 184	78 133 152 183	83 141 161 184	90 141 164 190	83 140 159 182
	5	82 136 152 167 187	82 136 152 167 187	82 136 152 167 187	72 143 153 162 184	82 136 152 167 187	89 134 145 166 191	81 136 152 167 187
Test-image2	2	73 134	73 134	73 134	75 134	73 134	74 134	73 134
	3	71 127 168	71 127 168	71 127 168	71 126 164	71 127 168	78 126 169	71 127 168
	4	69 120 142 178	69 120 142 178	69 120 142 178	57 121 142 175	69 120 142 178	70 122 145 182	68 119 141 177
	5	68 118 138 161 196	68 117 137 160 196	68 118 138 161 196	64 114 140 164 200	68 118 138 161 196	69 121 146 163 202	5 115 135 158 192
Test-image3	2	81 163	81 163	81 163	82 163	81 163	81 163	81 162
	3	73 135 179	73 135 179	73 135 179	72 137 177	73 135 179	71 135 179	73 134 178
	4	72 129 162 198	72 129 162 198	72 129 162 198	64 128 160 195	72 129 162 198	77 130 162 198	72 129 162 198
	5	70 123 148 173 204	70 123 148 173 204	70 124 149 174 204	80 122 147 176 197	70 124 149 174 204	71 124 148 173 203	70 123 148 172 203
Test-image4	2	95 170	95 170	95 170	94 170	95 170	94 170	95 170
	3	87 154 176	87 154 176	87 154 176	82 154 176	87 154 176	88 154 176	88 154 176
	4	71 130 158 177	71 130 158 177	86 152 171 187	68 149 169 184	86 152 171 187	75 131 158 177	70 129 158 177
	5	71 129 156 172 187	70 129 156 172 186	73 128 156 172 187	47 119 152 167 183	71 129 156 172 187	67 127 156 172 187	67 125 153 170 186
Test-image5	2	87 162	87 162	87 162	87 161	87 162	87 162	87 161
	3	83 150 185	83 150 185	83 150 185	75 150 185	83 150 185	83 150 185	83 150 184
	4	81 143 169 199	81 143 169 198	81 143 169 199	73 138 159 186	81 143 169 199	80 142 168 198	80 141 166 194
	5	77 134 155 177 205	77 134 155 177 205	77 134 155 177 205	60 120 152 176 203	77 134 155 177 205	76 134 155 177 204	74 133 155 177 205
Test-image6	2	78 146	78 146	78 146	77 146	78 146	78 146	78 146
	3	76 141 188	76 141 188	76 141 188	81 142 189	76 141 188	76 141 188	76 141 188
	4	74 133 161 196	74 133 161 196	74 133 161 196	69 138 164 197	74 133 161 196	73 134 162 196	74 133 161 196
	5	71 121 145 166 199	73 129 154 172 203	74 130 155 173 204	1 65 135 163 203	73 130 155 173 204	72 130 155 174 204	71 122 145 166 199
Test-image7	2	85 163	85 163	85 163	84 163	85 163	85 163	85 163
	3	74 144 180	74 144 180	74 144 180	70 145 182	74 144 180	74 144 180	74 144 179
	4	50 108 154 183	50 108 154 183	51 109 154 183	45 99 153 179	50 108 154 183	50 108 153 183	50 108 153 182
	5	50 106 149 174 199	50 106 149 174 199	51 106 149 174 199	66 114 152 176 204	50 106 149 174 199	49 105 150 175 201	50 106 148 174 199
Test-image8	2	91 165	91 165	91 165	91 165	91 165	91 165	91 165
	3	87 155 190	87 155 190	87 155 190	92 155 190	87 155 190	85 155 190	87 155 189
	4	84 146 174 201	84 146 174 201	84 146 174 201	68 140 171 199	84 146 174 201	86 147 175 201	84 146 173 199
	5	83 141 165 185 209	83 141 165 185 209	83 142 166 186 210	73 146 166 183 212	83 141 165 185 209	83 141 164 184 210	83 140 164 184 207
Test-image9	2	90 174	90 174	90 174	91 173	90 174	90 174	90 174
	3	89 165 206	89 165 206	89 165 206	91 163 203	89 165 206	90 165 206	89 165 206
	4	80 138 173 210	80 138 173 210	80 138 173 210	68 142 171 208	80 138 173 210	78 138 174 210	80 138 173 210
	5	55 88 120 156 200	80 137 168 196 220	80 137 168 196 221	1 82 144 173 215	80 137 168 196 221	74 137 169 197 223	78 137 168 196 221
Test-image10	2	82 153	82 153	82 153	81 153	82 153	82 153	82 153
	3	80 144 178	80 144 178	80 144 178	82 144 176	80 144 178	79 144 177	79 144 178
	4	76 129 156 181	76 129 156 181	76 129 156 181	82 134 163 190	76 129 156 181	76 128 156 181	76 128 155 181
	5	75 126 152 175 195	75 126 152 176 196	75 126 152 175 195	76 125 149 177 196	75 126 152 175 195	74 127 153 176 197	75 127 152 175 195

**Table 3:**
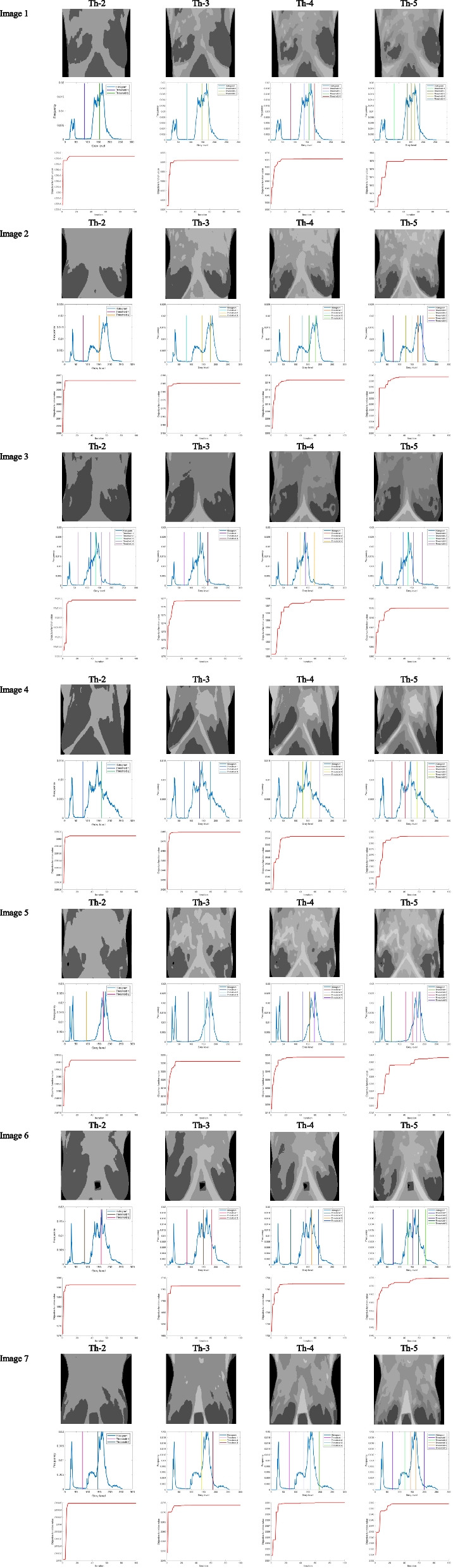
Results after applying IBWOA-Otsu’s method to the thermography images

Table 4 displays the fitness outcomes obtained from each level using the proposed IBWOA and other comparison algorithms, based on mean and standard deviation (STD). The most important values are shown in bold. A lower STD, in contrast to the mean, is preferred since it represents less variance between the outcomes provided by each strategy. As the situation becomes more complex, the STD rises along with the number of thresholds. According to the findings, the suggested IBWOA outperforms the original BWOA and WOA for all test images at all levels. It also performs better than the HHO, LSHADE, and SCA. For most test images, the LSHADE method is comparable to the proposed IBWOA and outperforms other algorithms. However, as seen in the table, the IBWOA excels in twenty-five different experiments. Among all competing algorithms, the WOA produces the least favorable results. The SCA, on the other hand, did not achieve any significant value in its mean findings.

**Table 4:** Comparison of Otsu’s fitness values

Test Image		HHO		LSHADE		WOA		SCA		BWOA		IBWOA	
	Th	Mean	Std	Mean	Std	Mean	Std	Mean	Std	Mean	Std	Mean	Std
Test-image1	2	1785.434	1.154 E − 12	1785.2251	1.153 E − 12	1785.2237	2.046 E − 03	**1785.4518**	2.626 E − 02	1785.2143	1.153 E − 12	**1785.4518**	1.153 E − 12
	3	1841.1385	5.746 E − 04	1840.9283	5.404 E − 03	1840.9284	2.126 E − 03	1838.8109	9.334	**1841.1569**	1.384 E − 12	**1841.1569**	1.384 E − 12
	4	1869.863	4.944	**1870.1607**	7.408 E − 03	1868.4571	6.877	1860.1368	12.06	1858.1466	9.228 E − 13	1869.8817	9.228 E − 13
	5	1881.8667	3.101	**1882.4086**	1.088	1879.6291	4.485	1867.7541	8.148	1872.6636	1.615 E − 12	1881.8855	1.889
Test-image2	2	1124.0974	9.237 E − 13	1123.8887	9.228 E − 13	1123.8867	5.996 E − 03	1123.8732	3.23 E − 02	1123.8772	9.228 E − 13	**1124.1087**	9.228 E − 13
	3	1278.7967	6.927 E − 13	1278.592	6.921 E − 13	1278.57	4.129 E − 02	1273.6181	26.06	1278.0514	6.921 E − 13	**1278.8095**	6.921 E − 13
	4	1316.8098	2.829 E − 02	**1316.823**	2.066 E − 02	1315.7015	6.458	1302.5139	16.93	1308.1765	1.153 E − 12	**1316.823**	1.153 E − 12
	5	1339.4283	2.509 E − 02	**1339.7271**	2.809 E − 02	1337.8614	6.233	1318.3765	11.71	1327.2993	7.287 E − 03	1339.4417	3.867
Test-image3	2	2297.6665	4.618 E − 13	2297.4576	4.614 E − 13	2297.4567	4.436 E − 04	2297.4463	1.477 0E − 02	2297.4427	4.614 E − 13	**2297.6895**	4.614 E − 13
	3	2483.922	2.498 E − 03	2483.7133	4.949 E − 03	2483.7075	1.475 E − 02	2482.9885	6.706 0E − 01	2483.0764	1.846 E − 12	**2483.9468**	1.846 E − 12
	4	2558.2559	1.377 E − 02	**2558.4827**	2.622 E − 03	2556.3088	12.63	2533.3446	32.27 9	2539.3152	9.228 E − 13	2558.2814	2.712 E − 04
	5	2589.619	4.059 E − 02	**2590.1193**	3.93 E − 02	2586.5636	10.01	2559.3378	23.89 4	2572.8335	2.826 E − 03	2589.6449	2.826 E − 03
Test-image4	2	**3194.5603**	1.847 E − 12	**3194.5603**	1.846 E − 12	3194.3179	5.453 E − 03	3194.275	7.493 5E − 02	**3194.5603**	1.846 E − 12	**3194.5603**	1.846 E − 12
	3	3233.75	1.847 E − 12	3233.5417	1.846 E − 12	3233.5378	9.298 E − 03	3229.3915	10.92 3	3230.5305	1.846 E − 12	**3233.7823**	1.846 E − 12
	4	3250.2614	8.19 E − 02	3250.1665	7.394 E − 02	3249.6748	2.809	3242.136	7.968 8	3244.4765	7.355 E − 02	**3250.2939**	4.009 E − 02
	5	**3265.5733**	4.333	**3265.5733**	3.336 E − 02	3264.1275	4.138	**3265.5733**	6.787 9	3253.4537	9.38 E − 04	3265.2603	3.364
Test-image5	2	1599.7184	1.154 E − 12	1599.5094	1.153 E − 12	1599.5085	1.153 E − 12	1599.4884	2.75 5E − 02	1599.4827	1.153 E − 12	**1599.7343**	1.153 E − 12
	3	1721.0525	4.618 E − 13	1720.8436	4.487 E − 04	1720.8422	1.077 E − 03	1719.6016	9.509 5E − 01	1719.6542	4.614 E − 13	**1721.0697**	4.614 E − 13
	4	1761.7358	6.813	**1762.1729**	1.702 E − 01	1761.0839	6.769	1747.3974	17.38 7	1754.7863	9.228 E − 13	1761.7534	6.803
	5	1789.1875	7.632	**1789.6789**	1.128	1788.5206	2.284	1763.1727	20.81 8	1778.7158	1.683	1789.2054	1.106
Test-image6	2	2115.5557	9.237 E − 13	2115.3469	9.228 E − 13	2115.3451	6.015 E − 03	2115.3041	5.88 0E − 02	2115.3166	9.228 E − 13	**2115.5768**	9.228 E − 13
	3	2275.2883	7.054 E − 03	2275.0799	4.944 E − 03	2275.0754	1.642 E − 02	**2275.311**	9.465 1E − 01	**2275.311**	4.614 E − 13	**2275.311**	4.614 E − 13
	4	2332.1222	7.28 E − 03	**2332.243**	3.842 E − 03	2330.596	9.66	2309.7143	26.07 2	2322.1505	9.228 E − 13	2332.1456	9.228 E − 13
	5	2353.1315	1.356 E − 01	2352.9722	1.655 E − 01	2352.6575	2.019	2332.4863	13.46 5	2341.9324	1.428 E − 01	**2353.155**	1.274 E − 01
Test-image7	2	3984.386	2.309 E − 12	3984.1775	2.307 E − 12	3984.1743	1.497 E − 02	3984.0798	1.217 2E − 01	3984.0613	2.307 E − 12	**3984.4258**	2.307 E − 12
	3	4082.276	9.237 E − 13	4082.065	2.082 E − 02	4082.0639	1.896 E − 02	4080.8133	7.64 6E − 01	4081.1187	9.228 E − 13	**4082.3168**	9.228 E − 13
	4	4149.577	2.282 E − 02	4149.375	1.243 E − 03	4149.3501	3.555 E − 02	4140.3731	18.57 3	4143.6414	2.967 E − 05	**4149.6185**	9.228 E − 13
	5	4184.8654	5.341 E − 02	**4185.0815**	1.343 E − 02	4184.0423	5.972	4158.2512	15.12 9	4170.3627	4.447 E − 03	4184.9072	1.876 E − 02
Test-image8	2	1829.1731	9.237 E − 13	1828.9642	9.228 E − 13	1828.9628	3.297 E − 03	1828.918	5.874 6E − 02	1828.9047	9.228 E − 13	**1829.1913**	9.228 E − 13
	3	1921.1073	9.237 E − 13	**1943.8849**	9.228 E − 13	1943.8718	2.746 E − 02	1939.6279	19.26 9	1939.5357	9.228 E − 13	1921.1265	9.228 E − 13
	4	1982.5222	1.255 E − 02	**1991.9176**	1.541 E − 02	1991.9147	2.218 E − 02	1968.6544	22.67 6	1982.2957	6.921 E − 13	1982.542	6.921 E − 13
	5	2016.536	5.925	**2017.1645**	7.706 E − 03	2015.1708	6.62	1987.7479	20.98 1	1999.03	4.257	2016.5561	2.081 E − 03
Test-image9	2	2748.4365	2.309 E − 12	2748.2275	2.307 E − 12	2748.2267	2.307 E − 12	2748.1954	6.084 5E − 02	2748.1979	2.307 E − 12	**2748.4639**	2.307 E − 12
	3	2853.1524	3.928 E − 03	2852.9446	1.846 E − 12	2852.94	1.529 E − 02	2845.9252	24.43 4	2851.82	1.846 E − 12	**2853.181**	1.846 E − 12
	4	2927.7783	1.718 E − 02	**2928.006**	2.825 E − 03	2925.8308	12.7	2910.6048	27.23 8	2914.3801	4.614 E − 13	2927.8075	4.614 E − 13
	5	2915.0768	9.276	**2956.7426**	2017 1635	2955.8165	4.736	2931.2131	11.74 3	2940.0162	2.275 E − 03	2915.106	2.275 E − 03
Test-image10	2	2096.8402	9.237 E − 13	2096.6315	9.228 E − 13	2096.6295	5.524 E − 03	2096.6025	4.882 3E − 02	2096.6271	9.228 E − 13	**2096.8612**	9.228 E − 13
	3	2186.028	3.483 E − 03	2185.8235	0	2185.8016	3.895 E − 02	2184.6715	7.521 9E − 01	2184.8148	0	**2186.0499**	0
	4	2217.15	1.646 E − 02	2217.1238	7.276 E − 03	2216.2134	5.288	2203.4938	13.84 6	2207.7278	9.228 E − 13	**2217.1722**	4.965 E − 03
	5	2240.1983	4.943 E − 02	2240.1215	2.91 E − 02	2239.4685	3.891	2214.9648	16.48 1	2222.641	2.509 E − 03	**2240.2207**	9.742 E − 03

In terms of the SSIM values based on the IBWOA algorithm and other comparison algorithms presented in Table 5, it can be seen that IBWOA generally performs better than the other algorithms. It shows significant results in all images at all levels except for image 7. The SCA exhibits higher SSIM values for two specific cases: test image-7 at level 2 and test image-5 at level 2. Furthermore, the WOA provides the best values in six different experiments, and LSHADE achieves one best value for test image-10 at level 2.

**Table 5:** Comparison of Otsu’s SSIM values

Test Image	Th	HHO		LSHADE		WOA		SCA		BWOA		IBWOA	
		Mean	Std	Mean	Std	Mean	Std	Mean	Std	Mean	Std	Mean	Std
Test-image1	2	0.8111	4.51 E − 16	0.8102	4.506 E − 16	0.8119	5.672 E − 04	0.8118	2.843 E − 03	**0.8124**	4.51 E − 16	0.8121	4.51 E − 16
	3	0.8158	5.637 E − 16	0.8149	6.218 E − 04	0.8171	8.459 E − 04	0.8138	8.665 E − 03	**0.8222**	5.637 E − 16	0.8208	3.466 E − 04
	4	0.838	1.395 E − 03	0.8381	9.439 E − 04	**0.8409**	6.739 E − 03	0.8336	1.357 E − 02	0.8386	2.255 E − 16	**0.8409**	2.169 E − 04
	5	0.8536	8.755 E − 03	0.8612	1.148 E − 03	0.8617	1.313 E − 02	0.8472	1.885 E − 02	0.8571	5.637 E − 16	**0.8649**	1.294 E − 02
Test-image2	2	0.8414	2.255 E − 16	0.8405	2.253 E − 16	0.8425	1.023 E − 03	0.8423	3.087 E − 03	**0.8435**	2.255 E − 16	0.843	2.255 E − 16
	3	0.8649	3.762 E − 04	0.864	4.37 E − 04	0.8661	3.068 E − 03	0.8646	1.055 E − 02	0.8676	4.51 E − 16	**0.8713**	4.51 E − 16
	4	**0.8749**	2.112 E − 03	0.8689	5.784 E − 04	**0.8749**	3.356 E − 03	0.8627	1.317 E − 02	0.8726	6.765 E − 16	**0.8749**	7.877 E − 04
	5	0.8763	2.677 E − 03	0.8781	3.358 E − 03	0.8784	7.108 E − 03	0.8746	1.939 E − 02	0.8801	1.026 E − 03	**0.8852**	2.954 E − 03
Test-image3	2	0.7904	4.51 E − 16	0.7896	4.506 E − 16	0.7913	4.71 E − 04	0.7925	2.674 E − 03	**0.7934**	4.51 E − 16	0.7932	4.51 E − 16
	3	0.8201	3.03 E − 04	0.8193	1.037 E − 03	0.8207	1.923 E − 03	0.8212	1.025 E − 02	0.8274	1.127 E − 16	**0.8281**	1.127 E − 16
	4	0.8295	1.868 E − 03	0.8305	1.775 E − 04	0.8318	3.95 E − 03	0.8223	1.483 E − 02	0.8348	4.51 E − 16	**0.8357**	4.51 E − 16
	5	0.8406	2.958 E − 03	0.8424	4.344 E − 04	0.844	3.385 E − 03	0.8312	1.686 E − 02	0.8443	5.18 E − 04	**0.8503**	3.483 E − 04
Test-image4	2	**0.7677**	1.127 E − 16	0.7661	1.126 E − 16	**0.7677**	4.339 E − 04	0.7666	2.392 E − 03	0.7672	1.127 E − 16	**0.7677**	1.127 E − 16
	3	0.8091	9.866 E − 05	0.8082	2.369 E − 04	**0.8101**	2.633 E − 04	0.8069	1.097 E − 02	0.807	5.637 E − 16	**0.8101**	5.637 E − 16
	4	0.8284	1.052 E − 02	0.8314	1.115 E − 02	0.8288	1.063 E − 02	0.8242	7.145 E − 03	0.8214	9.743 E − 03	**0.8344**	7.348 E − 03
	5	0.8387	1.061 E − 02	0.8421	2.57 E − 04	0.8431	2.046 E − 02	0.8462	3.007 E − 02	0.8371	1.125 E − 05	**0.8486**	2.265 E − 02
Test-image5	2	0.8342	3.382 E − 16	0.8333	3.379 E − 16	0.835	3.382 E − 16	0.8363	1.749 E − 03	**0.837**	3.382 E − 16	0.836	3.382 E − 16
	3	**0.8543**	5.637 E − 16	0.8488	5.748 E − 05	**0.8543**	6.875 E − 04	0.8537	5.733 E − 03	**0.8543**	5.637 E − 16	0.8539	5.637 E − 16
	4	0.8594	8.185 E − 04	0.8584	3.976 E − 03	0.8605	4.532 E − 04	0.8602	1.039 E − 02	0.8612	7.892 E − 16	**0.8619**	1.583 E − 04
	5	0.8807	2.603 E − 03	0.8814	4.083 E − 03	0.8882	8.7 E − 03	0.8738	1.89 E − 02	0.8804	7.365 E − 03	**0.8912**	5.073 E − 03
Test-image6	2	0.8193	3.382 E − 16	0.8184	3.379 E − 16	0.8201	2.142 E − 04	0.8192	1.536 E − 03	**0.8206**	3.382 E − 16	0.8201	3.382 E − 16
	3	0.8258	1.929 E − 04	0.8249	4.779 E − 04	0.8265	5.841 E − 04	0.8267	5.4 E − 03	**0.8299**	3.382 E − 16	0.8288	3.382 E − 16
	4	0.8298	6.726 E − 04	0.8297	4.401 E − 04	0.8332	1.058 E − 02	0.8312	7.446 E − 03	0.8326	3.382 E − 16	**0.8342**	3.382 E − 16
	5	0.841	3.694 E − 03	0.8433	3.513 E − 03	0.8412	3.578 E − 03	0.8408	9.594 E − 03	0.8434	2.757 E − 03	**0.8483**	2.304 E − 03
Test-image7	2	0.7556	2.255 E − 16	0.7548	2.253 E − 16	0.7564	1.324 E − 04	0.7559	1.971 E − 03	**0.7565**	2.255 E − 16	0.7564	2.255 E − 16
	3	0.7854	5.637 E − 16	0.7846	8.986 E − 05	0.7862	2.477 E − 05	**0.7869**	2.18 E − 03	0.7861	5.637 E − 16	0.7862	5.637 E − 16
	4	0.8174	2.316 E − 04	0.8164	1.737 E − 04	**0.8185**	4.041 E − 04	0.814	6.6 E − 03	0.8179	1.347 E − 04	0.8181	3.382 E − 16
	5	0.8242	8.201 E − 04	0.8233	4.136 E − 04	0.8259	4.483 E − 03	0.8246	6.648 E − 03	**0.8266**	1.28 E − 04	0.825	1.007 E − 04
Test-image8	2	0.8382	3.382 E − 16	0.8373	3.379 E − 16	0.839	2.644 E − 04	0.8387	2.265 E − 03	**0.84**	3.382 E − 16	0.8392	3.382 E − 16
	3	0.8367	4.51 E − 16	0.8358	4.506 E − 16	0.8378	9.971 E − 04	0.8362	6.554 E − 03	**0.8392**	4.51 E − 16	0.8387	4.51 E − 16
	4	0.8522	2.204 E − 03	0.8518	4.628 E − 04	0.8539	7.509 E − 04	0.8457	1.109 E − 02	**0.8545**	1.127 E − 16	0.8536	1.127 E − 16
	5	0.8635	2.804 E − 03	0.8645	3.01 E − 04	0.8678	9.106 E − 03	0.8531	1.308 E − 02	**0.868**	9.456 E − 05	0.8662	2.535 E − 04
Test-image9	2	0.8237	4.51 E − 16	0.8228	4.506 E − 16	0.8245	4.51 E − 16	0.824	3.125 E − 03	**0.8254**	4.51 E − 16	0.8249	4.51 E − 16
	3	0.8043	6.292 E − 04	0.8035	4.506 E − 16	0.8053	1.145 E − 03	0.8034	1.241 E − 02	0.8026	4.51 E − 16	**0.8058**	4.51 E − 16
	4	0.8316	6.667 E − 04	0.8312	1.585 E − 04	0.8326	3.399 E − 03	0.8208	1.176 E − 02	0.8311	4.51 E − 16	**0.835**	4.51 E − 16
	5	0.8384	1.127 E − 03	0.8391	5.231 E − 04	0.8429	1.174 E − 02	0.8345	1.183 E − 02	**0.8446**	2.176 E − 04	0.8435	3.416 E − 04
Test-image10	2	0.8399	4.51 E − 16	**0.8413**	4.506 E − 16	0.8407	3.099 E − 04	**0.8413**	1.203 E − 03	**0.8413**	4.51 E − 16	0.8407	4.51 E − 16
	3	0.8325	1.938 E − 04	0.8316	2.697 E − 04	0.8337	9.795 E − 04	0.8321	5.178 E − 03	**0.8356**	1.127 E − 16	**0.8356**	1.127 E − 16
	4	0.8465	5.293 E − 04	0.8458	3.624 E − 04	0.8475	1.882 E − 03	0.8387	8.657 E − 03	0.8463	5.637 E − 16	**0.8476**	9.166 E − 05
	5	0.8501	6.248 E − 04	0.8497	3.213 E − 04	0.8518	3.719 E − 04	0.8474	9.491 E − 03	0.8551	4.262 E − 05	**0.858**	2.467 E − 04

Image thresholding aims to produce more informative photographs using a limited number of thresholds. The PSNR (Peak Signal-to-Noise Ratio) is a performance metric frequently used to assess the quality of an output image compared to the original. While traditionally used for evaluating image quality, PSNR has been adapted to evaluate multi-dimensional signal functionality. In Table 6, a higher mean PSNR value indicates better image segmentation when considering the algorithm's thresholds. HHO and SCA provide only three and one best values, respectively, while LSHADE and WOA achieve five and seven best values, respectively. BWOA has limitations, yielding only three best values. In contrast, IBWOA delivers twenty-four best values out of forty experiments. Table 7 presents the FSIM (Feature Similarity Index) values for ten test images at 2, 3, 4, and 5 level thresholds, with IBWOA achieving the maximum number of best values.

**Table 6:** Comparison of Otsu’s PSNR values

Test Image		HHO		LSHADE		WOA		SCA		BWOA		IBWOA	
	Th	Mean	Std	Mean	Std	Mean	Std	Mean	Std	Mean	Std	Mean	Std
Test-image1	2	16.3172	0	16.3172	0	16.3171	2.993 E − 02	16.3179	1.5 E − 01	16.3072	1.351 E − 01	**16.3311**	1.404 E − 01
	3	17.4306	2.117 E − 02	17.46	6.418 E − 02	17.4628	5.358 E − 02	17.255	5.679 E − 01	**17.7474**	5.985 E − 01	17.6697	4.491 E − 01
	4	19.1508	1.657 E − 03	**19.183**	7.107 E − 02	19.0647	3.971 E − 01	18.6249	1.073	18.6145	1.128	19.151	1.882 E − 03
	5	20.3651	7.408 E − 02	20.3814	9.877 E − 02	20.3045	4.486 E − 01	19.5915	1.617	19.8151	1.208	**20.6755**	7.962 E − 01
Test-image2	2	17.0367	3.608 E − 15	17.0367	3.605 E − 15	17.0474	4.707 E − 02	17.0459	1.331 E − 01	17.0549	1.332 E − 01	**17.0728**	1.147 E − 01
	3	18.6582	0	18.6623	2.381 E − 02	18.6779	1.828 E − 01	18.6123	6.716 E − 01	18.7398	5.32 E − 01	**19.0199**	3.798 E − 01
	4	20.2314	5.662 E − 02	20.2552	4.129 E − 02	20.2543	3.706 E − 01	19.3468	1.149	20.0539	1.07	**20.2867**	1.15
	5	21.2997	2.319 E − 01	21.2516	2.598 E − 01	20.8729	1.966 E − 01	20.5207	1.573	20.9112	1.331	**21.3741**	1.024
Test-image3	2	14.7222	7.216 E − 15	14.7222	7.209 E − 15	14.7253	1.88 E − 02	14.774	1.083 E − 01	14.7783	1.465 E − 01	**14.8019**	6.479 E − 02
	3	17.9277	3.608 E − 15	17.9389	2.8 E − 02	17.9162	1.066 E − 01	17.9251	5.38 E − 01	18.2552	4.843 E − 01	**18.3253**	4.372 E − 01
	4	19.2945	1.082 E − 14	19.289	1.628 E − 02	19.2458	3.584 E − 01	18.4301	1.012	18.9216	8.78 E − 01	**19.4593**	8.422 E − 01
	5	20.4267	4.883 E − 02	20.4229	4.999 E − 02	20.2928	3.925 E − 01	19.1401	1.235	19.7776	1.124	**20.4515**	2.515 E − 02
Test-image4	2	**15.5918**	7.216 E − 15	5.5917	7.209 E − 15	15.5917	2.639 E − 02	15.5143	1.484 E − 01	15.4966	1.523 E − 01	**15.5918**	7.209 E − 15
	3	19.7142	7.216 E − 15	19.7141	6.885 E − 04	**19.7331**	3.745 E − 02	19.344	1.302	19.3782	1.048	19.7142	7.209 E − 15
	4	22.3003	6.814 E − 01	21.7684	1.02	21.8655	1.05	21.0251	1.263	20.4509	9.828 E − 01	**22.4174**	5.095 E − 01
	5	23.1758	4.876 E − 01	23.0487	1.744 E − 02	22.9917	5.847 E − 01	22.1876	1.205	21.7967	1.285	**23.1968**	6.271 E − 01
Test-image5	2	15.2716	9.02 E − 15	15.2716	9.011 E − 15	15.2715	9.011 E − 15	15.3534	9.16 E − 02	**15.356**	1.063 E − 01	15.2716	9.011 E − 15
	3	17.0752	0	17.0756	1.858 E − 03	17.0974	4.523 E − 02	17.2524	4.109 E − 01	17.2334	3.599 E − 01	**17.2646**	4.631 E − 01
	4	18.4472	1.763 E − 02	**18.6073**	2.876 E − 01	18.4375	2.131 E − 01	18.1152	9.24 E − 01	18.2917	7.794 E − 01	18.4503	9.734 E − 04
	5	20.5554	3.894 E − 01	20.7954	4.193 E − 01	20.8108	6.337 E − 01	19.2952	1.843	20.0109	1.349	**21.155**	7.397 E − 01
Test-image6	2	17.1194	1.443 E − 14	17.1194	1.442 E − 14	17.1213	1.205 E − 02	17.0824	7.241 E − 02	17.1147	6.183 E − 02	**17.1259**	4.767 E − 02
	3	18.2466	3.608 E − 15	18.2463	9.176 E − 04	18.2355	3.296 E − 02	18.16	3.067 E − 01	**18.3365**	2.437 E − 01	18.333	1.731 E − 01
	4	**19.8288**	1.443 E − 14	19.8208	3.382 E − 02	19.8112	2.015 E − 01	19.176	8.762 E − 01	19.7047	6.309 E − 01	**19.8288**	1.442 E − 14
	5	21.3268	2.992 E − 01	21.0649	4.494 E − 01	20.7089	3.202 E − 01	20.1507	9.913 E − 01	20.4963	7.76 E − 01	**21.5109**	1.794 E − 01
Test-image7	2	16.4397	0	16.4397	0	**16.4416**	1.207 E − 02	16.4144	8.345 E − 02	16.4154	8.368 E − 02	16.4397	0
	3	19.0146	1.443 E − 14	19.0184	1.239 E − 02	19.0099	1.649 E − 02	**19.0714**	1.738 E − 01	18.9538	1.359 E − 01	19.0146	1.442 E − 14
	4	21.2887	7.216 E − 15	21.295	1.353 E − 02	**21.337**	4.357 E − 02	20.8296	6.34 E − 01	21.168	5.96 E − 01	21.2887	7.209 E − 15
	5	22.304	1.355 E − 02	**22.3137**	2.168 E − 02	22.3082	1.301 E − 01	21.435	6.479 E − 01	21.9008	5.23 E − 01	22.3055	3.938 E − 03
Test-image8	2	16.3645	3.608 E − 15	16.3645	3.605 E − 15	**16.367**	1.552 E − 02	16.3478	1.306 E − 01	16.3625	1.355 E − 01	16.3645	3.605 E − 15
	3	18.0248	7.216 E − 15	18.0248	7.209 E − 15	18.0457	7.651 E − 02	17.9126	5.552 E − 01	18.0603	4.906 E − 01	**18.0931**	2.613 E − 01
	4	19.5487	1.443 E − 14	19.555	1.756 E − 02	**19.5875**	7.771 E − 02	18.6879	1.023	19.4497	7.755 E − 01	19.5487	1.442 E − 14
	5	20.4706	4.232 E − 02	20.4701	4.936 E − 02	20.39	1.752 E − 01	19.2733	1.185	20.0843	1.192	**20.4737**	1.422 E − 01
Test-image9	2	14.977	3.608 E − 15	14.977	3.605 E − 15	14.9769	3.605 E − 15	14.9524	1.593 E − 01	14.9854	1.551 E − 01	**14.9992**	9.489 E − 02
	3	16.0507	1.082 E − 14	16.0507	1.081 E − 14	**16.0571**	7.129 E − 02	15.8399	5.821 E − 01	15.8758	5.934 E − 01	16.0507	1.081 E − 14
	4	19.3928	1.082 E − 14	19.3951	9.517 E − 03	19.3255	5.205 E − 01	18.2339	1.3	18.9168	1.287	**19.4957**	4.741 E − 01
	5	20.1906	3.08 E − 02	20.2008	3.585 E − 02	**20.2182**	3.159 E − 01	19.3009	7.899 E − 01	19.8556	7.076 E − 01	20.1838	8.165 E − 02
Test-image10	2	**16.9947**	1.082 E − 14	**16.9947**	1.081 E − 14	16.9945	1.84 E − 02	16.9897	7.463 E − 02	16.9764	7.808 E − 02	**16.9947**	1.081 E − 14
	3	18.7358	7.216 E − 15	18.7396	1.555 E − 02	18.7694	7.978 E − 02	18.6657	3.922 E − 01	**18.8621**	3.57 E − 01	18.7358	7.209 E − 15
	4	20.5155	1.379 E − 02	**20.5231**	3.043 E − 02	20.4779	3.054 E − 01	19.4693	8.501 E − 01	20.0383	9.085 E − 01	20.5223	2.287 E − 02
	5	21.1605	2.425 E − 02	21.1568	3.563 E − 02	21.1679	9.561 E − 02	20.2306	1.018	20.5356	8.425 E − 01	**21.5638**	5.823 E − 01

**Table 7:** Comparison of Otsu’s FSIM values

Test Image		HHO		LSHADE		WOA		SCA		BWOA		IBWOA	
	Th	Mean	Std	Mean	Std	Mean	Std	Mean	Std	Mean	Std	Mean	Std
Test-image1	2	**0.8455**	5.637 E − 16	**0.8455**	5.632 E − 16	**0.8455**	5.856 E − 05	0.8454	3.607 E − 04	**0.8455**	2.684 E − 04	**0.8455**	5.632 E − 16
	3	0.8402	3.382 E − 16	0.8403	3.379 E − 16	0.8403	7.458 E − 05	0.84	2.485 E − 03	0.8408	2.727 E − 03	**0.841**	2.11 E − 03
	4	0.8432	4.116 E − 04	0.8432	5.632 E − 16	0.8431	2.294 E − 04	**0.8441**	3.738 E − 03	0.8428	3.071 E − 03	0.8435	4.363 E − 04
	5	0.8447	2.146 E − 03	**0.847**	3.379 E − 16	0.8458	2.993 E − 03	0.8445	4.08 E − 03	0.8469	3.857 E − 03	0.8468	3.181 E − 03
Test-image2	2	**0.8486**	4.51 E − 16	**0.8486**	4.506 E − 16	**0.8486**	9.774 E − 05	0.8485	5.848 E − 04	**0.8486**	2.728 E − 04	**0.8486**	4.506 E − 16
	3	0.858	2.025 E − 05	0.858	3.379 E − 16	0.8581	2.057 E − 04	0.8576	1.89 E − 03	0.8579	1.1 E − 03	**0.8583**	9.335 E − 04
	4	0.8429	3.743 E − 03	0.8429	3.379 E − 16	0.8434	2.611 E − 03	**0.8457**	8.817 E − 03	0.8454	6.348 E − 03	**0.8457**	7.215 E − 03
	5	0.8398	1.753 E − 03	0.8418	7.353 E − 04	0.8415	1.536 E − 03	0.8438	5.721 E − 03	0.8452	4.889 E − 03	**0.8473**	5.68 E − 03
Test-image3	2	0.8212	5.637 E − 16	0.8212	5.632 E − 16	0.8212	2.056 E − 05	**0.8214**	2.248 E − 04	0.8213	2.568 E − 04	0.8213	1.203 E − 04
	3	0.8174	2.885 E − 05	0.8174	5.632 E − 16	0.8174	1.635 E − 04	0.8179	1.864 E − 03	0.818	1.588 E − 03	**0.8184**	1.237 E − 03
	4	0.8163	1.588 E − 04	0.8163	5.632 E − 16	0.8163	4.509 E − 04	0.8174	2.849 E − 03	0.8184	2.774 E − 03	**0.8191**	2.583 E − 03
	5	0.8217	9.632 E − 04	**0.8222**	1.262 E − 04	0.8215	2.061 E − 03	0.817	3.341 E − 03	0.821	3.596 E − 03	0.8216	2.327 E − 04
Test-image4	2	**0.8751**	0	**0.8751**	0	**0.8751**	4.464 E − 05	0.8748	5.145 E − 04	0.8747	6.685 E − 04	**0.8751**	0
	3	0.8807	5.181 E − 06	0.8807	5.632 E − 16	0.8807	3.669 E − 05	**0.8809**	2.604 E − 03	0.8799	2.609 E − 03	0.8807	5.632 E − 16
	4	0.891	5.493 E − 03	0.8897	5.09 E − 03	0.8886	5.569 E − 03	0.886	4.603 E − 03	0.8813	3.393 E − 03	**0.8917**	2.814 E − 03
	5	**0.8949**	1.788 E − 04	0.8947	1.736 E − 05	0.894	3.101 E − 03	0.8899	5.027 E − 03	0.8883	5.869 E − 03	0.8947	8.344 E − 04
Test-image5	2	0.854	3.382 E − 16	0.854	3.379 E − 16	0.854	3.379 E − 16	**0.8544**	3.692 E − 04	0.8543	3.669 E − 04	0.8543	3.52 E − 04
	3	0.8451	3.382 E − 16	0.8451	3.379 E − 16	0.8452	1.254 E − 04	0.8464	1.763 E − 03	**0.8465**	2.097 E − 03	0.8461	1.351 E − 03
	4	0.8372	3.377 E − 04	0.8372	7.885 E − 16	0.8374	1.529 E − 03	**0.8406**	5.628 E − 03	0.8389	4.118 E − 03	0.8403	5.392 E − 03
	5	0.8409	1.644 E − 03	0.8433	3.353 E − 03	0.8446	4.383 E − 03	0.8424	6.149 E − 03	0.8408	5.019 E − 03	**0.8448**	6.06 E − 03
Test-image6	2	**0.8502**	7.892 E − 16	**0.8502**	7.885 E − 16	**0.8502**	4.316 E − 05	0.8498	5.776 E − 04	0.85	5.236 E − 04	**0.8502**	7.885 E − 16
	3	0.8464	5.499 E − 05	0.8464	4.506 E − 16	0.8464	4.01 E − 04	0.8485	4.23 E − 03	**0.8487**	2.77 E − 03	0.8484	1.817 E − 03
	4	0.8327	6.228 E − 04	0.8327	4.506 E − 16	0.833	3.039 E − 03	**0.8383**	8.172 E − 03	0.8341	6.351 E − 03	0.8354	6.393 E − 03
	5	0.8326	1.161 E − 03	0.8334	1.305 E − 03	0.8345	1.623 E − 03	**0.8357**	7.239 E − 03	0.8332	3.223 E − 03	0.8348	4.783 E − 03
Test-image7	2	**0.8373**	4.51 E − 16	**0.8373**	4.506 E − 16	**0.8373**	1.575 E − 05	0.8372	5.505 E − 04	**0.8373**	6.516 E − 04	**0.8373**	4.506 E − 16
	3	**0.8327**	5.637 E − 16	**0.8327**	5.632 E − 16	**0.8327**	3.004 E − 05	0.8317	9.393 E − 04	0.8321	7.908 E − 04	**0.8327**	5.632 E − 16
	4	0.8468	1.707 E − 04	0.8468	7.973 E − 05	**0.8471**	3.929 E − 04	0.8441	4.632 E − 03	0.8461	3.665 E − 03	0.8468	6.759 E − 16
	5	0.8474	6.171 E − 04	0.8474	1.04 E − 04	0.8476	5.294 E − 04	0.8471	4.424 E − 03	0.8471	3.679 E − 03	**0.8483**	2.476 E − 03
Test-image8	2	0.8421	4.51 E − 16	0.8421	4.506 E − 16	0.8422	3.077 E − 05	0.8422	6.279 E − 04	**0.8424**	8.76 E − 04	0.8422	1.787 E − 04
	3	0.814	5.637 E − 16	0.814	5.632 E − 16	0.814	1.109 E − 04	0.8145	5.633 E − 03	**0.8157**	5.409 E − 03	0.8152	5.325 E − 03
	4	0.8167	5.604 E − 04	0.8167	4.506 E − 16	0.8167	1.058 E − 04	0.8148	2.941 E − 03	0.8161	2.115 E − 03	**0.8168**	3.544 E − 04
	5	0.8207	1.092 E − 03	**0.8213**	1.482 E − 04	0.821	1.219 E − 03	0.8169	3.586 E − 03	0.8189	3.707 E − 03	0.8205	7.31 E − 04
Test-image9	2	**0.8518**	6.765 E − 16	**0.8518**	6.759 E − 16	**0.8518**	6.759 E − 16	**0.8518**	1.496 E − 04	**0.8518**	1.526 E − 04	**0.8518**	6.759 E − 16
	3	0.8275	4.48 E − 05	0.8275	5.632 E − 16	0.8275	1.041 E − 04	**0.8287**	6.552 E − 03	0.8277	3.067 E − 03	0.8286	2.149 E − 03
	4	0.8265	2.386 E − 04	0.8265	3.379 E − 16	**0.8268**	6.123 E − 04	0.8251	4.616 E − 03	0.8254	4.269 E − 03	**0.8268**	4.263 E − 03
	5	0.8283	4.242 E − 04	0.8283	3.78 E − 05	0.8286	8.653 E − 04	0.8256	5.249 E − 03	0.8271	4.633 E − 03	**0.8288**	4.138 E − 03
Test-image10	2	**0.842**	4.51 E − 16	**0.842**	4.506 E − 16	**0.842**	5.755 E − 05	**0.842**	2.279 E − 04	**0.842**	2.739 E − 04	**0.842**	4.506 E − 16
	3	0.8251	3.712 E − 05	0.8251	6.759 E − 16	**0.8252**	2.078 E − 04	0.8243	3.689 E − 03	0.8244	3.684 E − 03	0.8251	6.759 E − 16
	4	0.8203	3.112 E − 04	0.8204	7.885 E − 16	0.8206	8.766 E − 04	0.8219	4.152 E − 03	**0.8223**	3.944 E − 03	0.8216	3.085 E − 03
	5	0.8258	8.683 E − 04	0.8259	5.836 E − 05	0.8259	8.466 E − 04	0.8247	4.926 E − 03	0.8253	4.506 E − 03	**0.8273**	2.113 E − 03

#### Result and discussion: IBWOA-based Kapur results

The performance of the proposed thresholding strategy based on IBWOA applied to breast thermographic images using Kapur's entropy as the objective function is examined and discussed in this paragraph. Table 8 presents the threshold values derived from levels 2, 3, 4, and 5 using various algorithms, including HHO, LSHADE, WOA, SCA, and BWOA, as discussed in the previous section. The quality of the thresholded images employing Kapur's method is assessed and compared using thresholding values, SSIM, PSNR, and FSIM. Each test image includes four thresholding levels *[Th* = *2, 3, 4, 5]*, as was done with Otsu's algorithm. Table 9 consists of three rows: the first row contains segmented images after applying Kapur’s entropy, the second row shows the distribution of the best-selected thresholding values generated by IBWOA plotted on the histogram of each test image, and the third row provides convergence graphs. The mean and standard deviation of fitness, SSIM, PSNR, and FSIM are shown in Tables 10, 11, 12, and 13, respectively.

**Table 8:** Kapur’s optimal thresholds values

Test Image	Th	HHO	LSHADE	WOA	SCA	SSA	BWOA	IBWOA
Test-image1	2	52 115	52 115	52 115	52 116	52 115	54 115	52 115
	3	52 115 213	52 115 213	52 115 213	56 106 210	52 115 213	52 115 213	52 115 213
	4	52 115 181 215	52 115 181 215	52 115 181 215	51 114 185 228	52 115 181 215	52 115 181 215	52 115 181 215
	5	48 80 115 181 215	19 52 115 180 215	52 115 151 184 219	18 57 110 179 233	52 115 151 184 219	52 84 115 181 215	19 52 115 183 215
Test-image2	2	105 176	105 176	105 176	106 176	105 176	105 176	105 176
	3	105 158 196	105 158 196	105 158 196	101 155 190	105 158 196	105 158 196	105 158 196
	4	98 127 158 196	44 87 158 196	46 87 126 179	48 88 152 197	44 87 158 196	44 87 158 196	44 87 158 196
	5	44 87 123 158 196	44 87 123 158 196	44 87 124 158 196	44 87 125 169 194	44 87 123 158 196	44 87 123 158 196	44 87 123 158 196
Test-image3	2	127 189	127 189	127 189	128 191	127 189	127 189	127 189
	3	105 155 201	105 155 201	105 155 201	106 154 205	105 155 201	45 86 167	105 155 201
	4	45 86 142 196	45 86 146 196	45 86 146 196	45 86 147 199	42 90 138 205	45 86 146 196	45 86 146 196
	5	44 86 128 167 208	16 43 87 146 196	45 86 128 167 208	31 68 112 163 228	45 86 128 167 208	45 86 128 167 208	45 86 128 167 208
Test-image4	2	42 128	42 128	42 128	42 130	42 128	42 128	42 128
	3	44 101 137	44 101 137	44 97 137	44 104 136	44 97 137	44 101 137	44 97 137
	4	44 97 137 206	44 97 137 206	44 101 137 206	50 101 135 204	44 97 137 206	44 101 137 206	44 97 137 206
	5	44 96 134 160 206	16 49 92 133 213	44 88 130 160 206	16 46 98 140 207	44 96 134 160 206	44 93 134 160 206	16 42 107 137 206
Test-image5	2	121 203	121 203	121 203	121 203	121 203	121 203	121 203
	3	41 109 203	41 109 203	41 109 203	37 108 205	41 109 203	41 109 203	41 109 203
	4	41 109 157 204	41 109 157 204	41 109 158 204	48 109 152 209	41 109 157 204	41 109 157 204	41 109 157 204
	5	39 72 111 156 204	39 72 111 157 204	38 72 111 157 204	38 79 111 156 209	41 72 111 156 204	39 72 111 157 203	39 72 109 157 204
Test-image6	2	137 190	137 190	137 190	138 192	137 190	137 190	137 190
	3	46 96 190	22 137 190	46 96 190	22 131 189	22 137 190	22 137 190	22 137 190
	4	46 96 143 192	46 96 143 192	46 96 143 192	45 91 131 187	46 96 143 192	46 96 143 192	46 96 143 192
	5	45 96 143 187 221	22 45 96 143 190	48 96 143 187 221	21 39 92 134 199	46 96 143 187 221	22 46 96 143 192	22 50 96 145 193
Test-image7	2	34 114	34 114	146 200	147 199	146 200	34 114	146 200
	3	34 114 198	37 114 198	41 114 198	33 116 195	34 114 198	34 114 198	34 114 198
	4	34 114 155 200	34 114 154 200	38 114 155 200	39 114 149 196	34 114 155 200	34 114 155 200	34 114 155 200
	5	30 69 114 154 200	30 69 114 154 200	30 72 114 155 200	27 52 113 152 204	30 69 114 155 200	30 69 114 155 200	30 69 114 155 200
Test-image8	2	148 208	148 208	148 208	148 210	148 208	148 208	148 208
	3	49 109 208	49 109 208	49 109 208	50 109 201	49 109 208	49 109 208	49 109 208
	4	49 108 160 210	49 108 159 208	49 108 160 210	50 110 166 209	49 108 160 208	49 108 159 208	49 108 159 208
	5	49 107 144 174 211	48 108 158 202 230	48 80 110 160 210	50 104 143 177 219	49 108 158 202 230	49 107 144 174 211	48 80 110 161 208
Test-image9	2	142 214	142 214	142 214	141 214	142 214	142 214	142 214
	3	47 106 177	132 177 220	132 177 220	133 176 219	132 177 220	131 175 218	47 106 177
	4	47 106 163 217	47 106 163 217	47 106 163 217	54 100 155 216	47 106 163 217	47 106 163 217	47 106 163 217
	5	47 93 139 181 220	18 47 106 164 217	51 93 139 181 220	46 92 140 181 219	51 93 139 181 220	18 47 106 163 217	47 93 139 180 218
Test-image10	2	148 210	148 210	148 210	143 211	148 210	148 210	148 210
	3	112 158 210	22 148 210	112 158 210	87 149 222	22 148 210	22 148 210	22 148 210
	4	50 102 157 210	50 102 157 210	51 102 157 210	48 101 168 208	50 102 157 210	50 102 157 210	50 102 157 210
	5	44 102 133 163 210	50 102 133 163 210	22 65 102 157 210	22 63 93 143 219	22 50 102 157 210	50 102 133 163 210	22 50 102 157 210

**Table 9:**
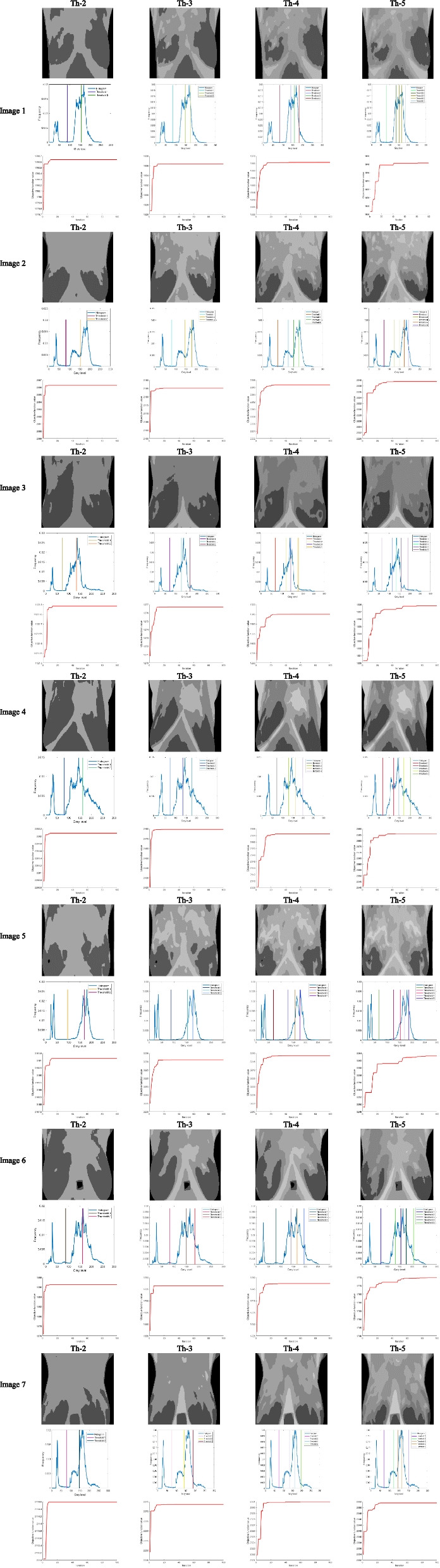
Results after applying IBWOA- Kapur’s method to the thermography images

**Table 10:** Objective function results for IBWOA- Kapur’s method

Test Image		HHO		LSHADE		WOA		SCA		BWOA		IBWOA	
	Th	Mean	Std	Mean	Std	Mean	Std	Mean	Std	Mean	Std	Mean	Std
Test-image1	2	16.7428	8.198 E − 02	16.7793	1.364 E − 02	16.7554	7.25 E − 02	16.7793	1.364 E − 02	16.7888	7.251 E − 03	**16.84**	0
	3	21.7863	1.237 E − 01	21.6267	9.549 E − 02	21.7709	1.588 E − 01	21.6267	9.549 E − 02	**21.8041**	2.097 E − 01	**21.804**	1.081 E − 14
	4	26.2187	1.877 E − 01	25.7747	2.288 E − 01	26.2158	1.19 E − 01	25.7747	2.288 E − 01	25.9025	1.24 E − 01	**26.232**	3.901 E − 02
	5	30.1912	1.967 E − 01	29.4144	6.008 E − 01	**30.2579**	1.274 E − 01	29.4144	6.008 E − 01	**30.2579**	1.749 E − 01	30.224	1.841 E − 01
Test-image2	2	17.0406	0	17.0316	8.109 E − 03	17.0402	2.544 E − 03	17.0316	8.109 E − 03	17.0385	3.065 E − 03	**17.085**	0
	3	21.4526	8.579 E − 02	21.3924	7.404 E − 02	21.4679	8.129 E − 02	21.3924	7.404 E − 02	21.3701	1.3 E − 01	**21.473**	3.883 E − 02
	4	25.726	2.217 E − 01	25.4137	1.704 E − 01	25.7239	1.511 E − 01	25.4137	1.704 E − 01	25.5786	1.524 E − 01	**25.805**	3.386 E − 03
	5	**29.8551**	1.929 E − 01	**29.8551**	3.968 E − 01	29.8409	1.57 E − 01	28.9793	3.968 E − 01	29.4668	2.012 E − 01	29.829	5.877 E − 03
Test-image3	2	17.5237	1.443 E − 14	17.5216	1.661 E − 03	17.5237	1.442 E − 14	17.5216	1.661 E − 03	17.5231	5.62 E − 04	**17.563**	5.439 E − 05
	3	21.8752	4.366 E − 03	21.8316	2.935 E − 02	**21.876**	3.185 E − 03	21.8316	2.935 E − 02	21.8417	6.232 E − 02	21.872	2.192 E − 03
	4	26.2738	2.481 E − 01	26.0502	2.216 E − 01	**26.3672**	1.225 E − 01	**26.3672**	2.216 E − 01	26.1686	2.358 E − 01	26.348	8.596 E − 04
	5	30.398	2.517 E − 01	29.7231	2.933 E − 01	**30.4471**	1.693 E − 01	29.7231	2.933 E − 01	30.1211	1.462 E − 01	30.393	2.761 E − 02
Test-image4	2	16.0854	3.608 E − 15	16.0795	5.554 E − 03	16.0854	3.13 E − 04	**16.139**	5.554 E − 03	16.084	1.57 E − 03	**16.139**	3.605 E − 15
	3	20.6125	1.877 E − 02	20.5377	3.634 E − 02	20.5968	3.521 E − 02	20.5377	3.634 E − 02	20.5458	1.004 E − 01	**20.628**	3.001 E − 04
	4	25.0228	1.919 E − 01	24.6552	2.178 E − 01	**25.0695**	3.424 E − 02	24.6552	2.178 E − 01	24.7639	1.768 E − 01	25.046	9.063 E − 03
	5	28.9393	8.356 E − 02	28.1816	5.686 E − 01	**28.9546**	7.853 E − 02	28.1816	5.686 E − 01	28.6207	1.393 E − 01	28.87	2.9 E − 02
Test-image5	2	17.1908	1.082 E − 14	17.173	1.724 E − 02	17.1908	1.081 E − 14	17.173	1.724 E − 02	17.1831	6.9 E − 03	**17.233**	1.081 E − 14
	3	21.6192	8.591 E − 02	21.5649	6.617 E − 02	21.5737	9.25 E − 02	21.5649	6.617 E − 02	21.6222	3.995 E − 02	**21.67**	1.714 E − 03
	4	**26.0647**	4.936 E − 02	25.8094	1.386 E − 01	26.0392	5.373 E − 02	25.8094	1.386 E − 01	25.9352	7.445 E − 02	26.027	2.499 E − 03
	5	30.0642	2.331 E − 01	29.4174	6.564 E − 01	**30.0734**	1.413 E − 01	29.4174	6.564 E − 01	29.7698	1.092 E − 01	30.06	3.341 E − 02
Test-image6	2	17.3937	1.053 E − 01	17.4158	3.741 E − 03	17.4192	7.209 E − 15	17.4158	3.741 E − 03	17.341	1.708 E − 01	**17.46**	7.209 E − 15
	3	21.6058	1.142 E − 01	21.6022	8.045 E − 02	21.6679	1.548 E − 01	21.6022	8.045 E − 02	21.8231	7.032 E − 02	**21.835**	7.396 E − 02
	4	**26.1896**	3.106 E − 02	25.7806	2.287 E − 01	26.131	1.193 E − 01	25.7806	2.287 E − 01	26.0256	7.092 E − 02	26.146	1.121 E − 02
	5	30.3241	3.164 E − 01	29.731	2.635 E − 01	**30.4921**	2.697 E − 01	29.731	2.635 E − 01	30.2591	1.72 E − 01	30.465	1.695 E − 01
Test-image7	2	16.7133	9.873 E − 03	16.716	1.449 E − 02	16.7197	1.293 E − 02	16.716	1.449 E − 02	16.7098	4.271 E − 03	**16.772**	1.407 E − 02
	3	**21.4905**	7.216 E − 15	**21.4905**	2.877 E − 02	21.4878	6.139 E − 03	21.4346	2.877 E − 02	21.4569	1.851 E − 02	**21.491**	3.88 E − 05
	4	25.9305	5.446 E − 02	25.7784	4.977 E − 02	**25.9483**	3.947 E − 02	25.7784	4.977 E − 02	25.8763	4.128 E − 02	25.919	2.663 E − 02
	5	**30.2263**	1.157 E − 02	29.7545	1.697 E − 01	30.214	3.009 E − 02	29.7545	1.697 E − 01	29.9477	8.844 E − 02	30.145	5.771 E − 04
Test-image8	2	16.9369	1.193 E − 01	16.9924	5.203 E − 03	17.0009	7.209 E − 15	16.9924	5.203 E − 03	16.981	6.506 E − 02	**17.045**	7.209 E − 15
	3	21.5813	1.445 E − 01	**21.6633**	6.335 E − 02	21.6277	9.69 E − 02	**21.6633**	6.335 E − 02	21.6084	3.606 E − 02	**21.663**	2.219 E − 04
	4	26.2017	3.436 E − 01	25.836	7.47 E − 01	**26.283**	4.365 E − 04	25.836	7.47 E − 01	26.1208	6.027 E − 02	26.236	1.619 E − 05
	5	30.0876	3.543 E − 02	29.3652	8.71 E − 01	**30.1013**	3.684 E − 02	29.3652	8.71 E − 01	29.8907	1.003 E − 01	30.025	2.012 E − 02
Test-image9	2	16.9909	7.32 E − 02	16.9957	7.581 E − 03	17.0033	1.442 E − 14	16.9957	7.581 E − 03	16.866	2.029 E − 01	**17.048**	1.442 E − 14
	3	21.1929	8.833 E − 03	21.1542	3.744 E − 02	21.1932	1.133 E − 02	21.1542	3.744 E − 02	21.1613	2.746 E − 02	**21.195**	8.407 E − 03
	4	**25.6298**	6.997 E − 02	25.4013	1.681 E − 01	25.6035	6.999 E − 02	25.4013	1.681 E − 01	25.3933	1.558 E − 01	25.607	1.02 E − 03
	5	29.6428	1.425 E − 01	29.119	4.25 E − 01	**29.6873**	1.052 E − 01	29.119	4.25 E − 01	29.5513	9.644 E − 02	29.623	5.301 E − 02
Test-image10	2	17.2276	1.375 E − 01	17.243	8.493 E − 03	17.2509	3.605 E − 15	17.243	8.493 E − 03	17.1049	2.672 E − 01	**17.293**	3.605 E − 15
	3	21.6461	2.062 E − 02	21.5201	7.608 E − 02	21.6483	1.694 E − 02	21.5201	7.608 E − 02	21.6333	4.642 E − 02	**21.656**	2.985 E − 02
	4	**26.3531**	7.957 E − 02	25.9285	2.066 E − 01	26.3085	9.663 E − 02	25.9285	2.066 E − 01	26.0804	1.611 E − 01	26.331	3.605 E − 15
	5	30.4851	3.305 E − 01	29.3474	1.114	30.4251	3.401 E − 01	29.3474	1.114	30.2271	2.419 E − 01	**30.634**	1.648 E − 01

**Table 11:** Comparison of Kapur’s method SSIM values

Test Image		HHO		LSHADE		WOA		SCA		BWOA		IBWOA	
	Th	Mean	Std	Mean	Std	Mean	Std	Mean	Std	Mean	Std	Mean	Std
Test-image1	2	0.8508	1.518 E − 03	0.8624	7.638 E − 03	0.8547	1.834 E − 02	0.8634	1.517 E − 03	**0.8657**	2.068 E − 02	**0.8657**	6.759 E − 16
	3	0.86	6.41 E − 03	0.8609	1.678 E − 03	0.862	5.76 E − 03	0.8564	6.404 E − 03	**0.8674**	6.727 E − 03	0.8622	6.044 E − 04
	4	0.8537	3.584 E − 02	0.8544	2.201 E − 02	0.8572	2.425 E − 02	0.8609	3.581 E − 02	**0.899**	1.853 E − 02	**0.899**	2.538 E − 02
	5	0.869	3.08 E − 02	0.8754	2.09 E − 02	0.8754	2.277 E − 02	0.8883	3.077 E − 02	**0.9041**	2.587 E − 02	0.8902	2.502 E − 02
Test-image2	2	0.8037	7.775 E − 03	0.8037	8.724 E − 05	0.8037	8.724 E − 05	0.8034	7.768 E − 03	0.8022	2.253 E − 16	**0.8045**	2.253 E − 16
	3	0.8188	4.402 E − 02	0.8039	2.253 E − 16	0.8103	1.88 E − 02	0.8109	4.398 E − 02	**0.8293**	2.561 E − 02	**0.8293**	2.72 E − 02
	4	0.8646	2.625 E − 02	0.8637	9.059 E − 03	**0.8687**	1.514 E − 02	0.8512	2.623 E − 02	0.8574	9.155 E − 03	0.865	6.631 E − 03
	5	0.9039	2.516 E − 02	0.9044	5.301 E − 03	0.898	1.585 E − 02	0.8948	2.514 E − 02	0.8949	7.497 E − 03	**0.9055**	5.081 E − 03
Test-image3	2	0.6176	9.978 E − 03	0.6176	6.543 E − 05	0.6176	0	0.6176	9.969 E − 03	**0.621**	0	0.6182	6.543 E − 05
	3	0.7846	1.972 E − 02	0.7826	3.985 E − 03	0.7832	5.839 E − 03	0.7787	1.971 E − 02	0.771	7.997 E − 03	**0.7885**	1.482 E − 02
	4	0.8208	2.091 E − 02	0.819	3.621 E − 03	0.8225	7.927 E − 03	0.8227	2.089 E − 02	0.811	6.084 E − 03	**0.8263**	5.11 E − 03
	5	0.8515	2.877 E − 02	0.8497	4.543 E − 03	0.8523	5.831 E − 03	0.8484	2.875 E − 02	0.8539	1.139 E − 02	**0.8565**	1.314 E − 02
Test-image4	2	0.8168	1.419 E − 03	0.8168	4.506 E − 16	0.8168	9.946 E − 06	0.818	1.418 E − 03	**0.819**	4.506 E − 16	0.8176	4.506 E − 16
	3	0.8327	5.76 E − 03	**0.8359**	8.497 E − 03	0.8269	1.308 E − 02	0.8322	5.755 E − 03	0.8309	7.167 E − 03	**0.8359**	1.253 E − 04
	4	**0.8533**	3.285 E − 02	0.8318	7.885 E − 16	0.8315	4.001 E − 04	0.8376	3.282 E − 02	**0.8533**	1.999 E − 03	**0.8533**	2.572 E − 02
	5	0.8425	4.547 E − 02	0.8443	2.354 E − 02	0.8611	4.517 E − 02	**0.8909**	4.543 E − 02	0.8904	2.464 E − 02	0.8631	4.484 E − 02
Test-image5	2	0.8342	9.106 E − 03	0.8342	6.759 E − 16	0.8342	6.759 E − 16	0.8295	9.097 E − 03	0.8322	6.759 E − 16	**0.8355**	2.534 E − 03
	3	0.859	6.447 E − 03	0.8597	1.084 E − 02	0.8534	1.245 E − 02	0.8624	6.441 E − 03	**0.8677**	1.12 E − 02	0.8666	5.176 E − 04
	4	0.8919	1.38 E − 02	0.8925	4.664 E − 04	0.8918	2.9 E − 03	0.8822	1.379 E − 02	0.8899	3.188 E − 03	**0.8936**	1.354 E − 03
	5	**0.8994**	1.405 E − 02	0.8981	1.212 E − 03	0.8965	3.373 E − 03	0.8876	1.404 E − 02	0.8905	5.135 E − 03	0.8991	5.061 E − 03
Test-image6	2	0.646	1.008 E − 02	0.6617	0	0.6617	0	0.6609	1.007 E − 02	0.6169	6.48 E − 02	**0.6624**	6.733 E − 04
	3	0.8033	2.437 E − 02	0.8038	2.871 E − 04	0.8033	2.064 E − 03	**0.8076**	2.435 E − 02	0.801	2.444 E − 03	0.8072	1.487 E − 02
	4	0.8576	1.468 E − 02	0.8564	2.763 E − 04	0.8545	2.224 E − 02	0.8582	1.467 E − 02	0.8595	7.482 E − 03	**0.8602**	1.417 E − 02
	5	0.8919	3.185 E − 02	0.8873	3.89 E − 02	0.9204	2.463 E − 02	0.9036	3.182 E − 02	0.923	3.705 E − 02	**0.9305**	5.882 E − 03
Test-image7	2	0.7727	6.01 E − 02	0.7488	5.751 E − 02	0.7249	6.038 E − 02	0.7423	6.004 E − 02	**0.7883**	4.239 E − 02	0.7862	2.508 E − 02
	3	0.7805	4.203 E − 03	0.7805	6.214 E − 05	0.7811	5.766 E − 04	0.7823	4.199 E − 03	**0.7829**	3.379 E − 16	0.7823	6.679 E − 03
	4	0.8159	5.489 E − 03	0.8183	2.098 E − 03	**0.8202**	3.455 E − 03	0.8112	5.484 E − 03	0.8182	4.29 E − 03	**0.8202**	1.234 E − 03
	5	0.8344	9.338 E − 03	0.8338	2.112 E − 04	0.8327	1.345 E − 03	0.8234	9.329 E − 03	0.8267	6.276 E − 04	**0.8355**	5.691 E − 04
Test-image8	2	**0.7068**	1.384 E − 02	0.6706	2.253 E − 16	0.6706	2.253 E − 16	0.6724	1.383 E − 02	0.681	6.736 E − 02	0.6893	5.104 E − 02
	3	0.8147	2.815 E − 02	0.8107	4.616 E − 03	0.8148	1.445 E − 02	0.8141	2.813 E − 02	0.8084	8.549 E − 03	**0.8199**	1.062 E − 02
	4	0.8631	1.663 E − 02	0.8642	6.568 E − 04	0.8636	1.937 E − 05	0.8529	1.662 E − 02	0.8624	3.663 E − 03	**0.8652**	7.478 E − 04
	5	0.8694	9.788 E − 03	**0.8706**	5.991 E − 03	0.8652	5.771 E − 03	0.8636	9.779 E − 03	0.8627	5.346 E − 03	**0.8706**	5.39 E − 03
Test-image9	2	0.6364	1.706 E − 02	0.6368	2.253 E − 16	0.6368	2.253 E − 16	0.6271	1.705 E − 02	0.6327	2.477 E − 03	**0.6418**	2.614 E − 02
	3	0.7955	5.756 E − 02	0.7538	4.369 E − 02	0.7679	5.188 E − 02	0.7858	5.751 E − 02	0.7865	5.841 E − 02	**0.8194**	5.124 E − 02
	4	**0.8507**	1.072 E − 02	0.8292	5.013 E − 04	0.8228	8.702 E − 03	0.8181	1.071 E − 02	**0.8507**	1.998 E − 02	0.8328	1.406 E − 02
	5	0.8524	2.656 E − 02	0.8557	1.639 E − 02	0.8579	1.878 E − 02	0.8673	2.654 E − 02	**0.8873**	1.848 E − 02	0.8693	2.035 E − 02
Test-image10	2	0.6234	1.454 E − 02	0.6176	1.126 E − 16	0.6176	1.126 E − 16	0.6249	1.453 E − 02	0.5582	3.199 E − 02	**0.6255**	1.453 E − 02
	3	0.8066	3.901 E − 02	0.8073	2.117 E − 02	0.8121	1.241 E − 02	0.7938	3.898 E − 02	0.7862	1.388 E − 02	**0.8126**	5.366 E − 03
	4	0.8705	1.551 E − 02	0.8687	5.632 E − 16	**0.8733**	1.133 E − 02	0.8582	1.55 E − 02	0.8697	6.816 E − 03	0.8722	1.145 E − 02
	5	0.9035	2.273 E − 02	0.9048	2.798 E − 02	0.9017	2.791 E − 02	0.9032	2.271 E − 02	0.915	2.797 E − 02	**0.9298**	7.566 E − 03

**Table 12:** Comparison of Kapur’s method PSNR values

Test Image		HHO		LSHADE		WOA		SCA		BWOA		IBWOA	
	Th	Mean	Std	Mean	Std	Mean	Std	Mean	Std	Mean	Std	Mean	Std
Test-image1	2	15.3312	6.428 E − 02	15.2996	3.489 E − 02	15.3237	6.094 E − 02	15.2972	1.357 E − 01	15.2972	1.357 E − 01	**15.597**	2.096 E − 01
	3	15.4852	3.56 E − 01	15.4795	1.534 E − 01	15.4731	1.224 E − 01	15.3174	5.107 E − 01	15.3174	5.107 E − 01	**15.763**	1.992 E − 01
	4	16.4424	3.211 E − 01	16.4996	2.495 E − 01	16.8936	1.167	16.2215	1.802	16.2215	1.802	**16.903**	1.301
	5	17.7275	1.98	19.5495	2.279	**19.7523**	2.146	17.5948	2.371	17.5948	2.371	17.103	2.287
Test-image2	2	15.6426	9.02 E − 15	15.6482	3.332 E − 02	15.6482	3.332 E − 02	15.6645	1.36 E − 01	15.6645	1.36 E − 01	**15.955**	9.011 E − 15
	3	15.9272	3.159 E − 01	16.0763	3.605 E − 15	16.0158	2.387 E − 01	15.7978	7.578 E − 01	15.7978	7.578 E − 01	**16.398**	2.09 E − 01
	4	16.8743	2.081	16.3558	1.705	**16.9257**	2.04	16.1628	2.037	16.1628	2.037	15.985	1.474
	5	21.4714	1.011	21.5142	9.978 E − 01	20.7074	2.154	18.4704	2.335	18.4704	2.335	**22.129**	9.978 E − 01
Test-image3	2	**12.9181**	9.02 E − 15	12.6654	2.488 E − 03	**12.9181**	9.011 E − 15	12.6621	1.796 E − 01	12.6621	1.796 E − 01	**12.918**	9.011 E − 15
	3	17.1365	7.311 E − 01	17.3382	3.912 E − 01	17.2641	5.324 E − 01	16.0957	1.326	16.0957	1.326	**17.741**	3.952 E − 01
	4	**18.3924**	4.728 E − 01	17.8905	2.819 E − 01	18.024	2.273 E − 01	17.3028	1.389	17.3028	1.389	18.255	2.962 E − 01
	5	20.4212	6.04 E − 01	**21.064**	1.018 E − 01	**21.064**	6.34 E − 01	18.0498	1.712	18.0498	1.712	**21.064**	3.723 E − 01
Test-image4	2	15.2945	5.412 E − 15	15.2945	5.407 E − 15	15.2946	4.967 E − 04	15.4604	1.902 E − 01	15.4604	1.902 E − 01	**15.6**	5.407 E − 15
	3	16.7549	4.784 E − 01	16.6708	5.664 E − 01	16.3539	9.316 E − 01	16.6305	4.76 E − 01	16.6305	4.76 E − 01	**17.257**	2.849 E − 03
	4	17.0794	4.223 E − 02	17.0992	3.605 E − 15	17.0922	1.365 E − 02	16.8082	1.208	16.8082	1.208	**17.434**	5.057 E − 02
	5	18.9877	2.006	**20.1988**	1.77	19.6513	1.883	18.9845	1.933	18.9845	1.933	17.943	2.034
Test-image5	2	14.8944	1.803 E − 15	14.8944	1.802 E − 15	14.8944	1.802 E − 15	14.8591	1.115 E − 01	14.8591	1.115 E − 01	**15.192**	8.738 E − 02
	3	15.2683	1.573	15.1704	1.521	**16.053**	1.739	14.4221	5.18 E − 01	14.4221	5.18 E − 01	14.574	1.797
	4	19.5623	8.598 E − 01	19.7105	3.706 E − 02	19.6396	2.703 E − 01	18.3104	1.691	18.3104	1.691	**20.099**	7.306 E − 02
	5	**20.4751**	5.629 E − 01	20.3709	4.802 E − 01	20.3094	6.9 E − 01	18.9084	1.539	18.9084	1.539	20.266	3.813 E − 01
Test-image6	2	12.4739	1.423	12.8189	1.802 E − 15	12.8189	1.802 E − 15	12.7981	1.71 E − 01	12.7981	1.71 E − 01	**13.075**	1.802 E − 15
	3	**16.7965**	1.859	15.1319	1.426	16.3519	1.887	14.5202	1.197	14.5202	1.197	14.718	2.176
	4	20.0305	8.749 E − 02	20.0396	2.092 E − 02	19.1921	1.998	17.3197	2.938	17.3197	2.938	**20.445**	2.092 E − 02
	5	20.6672	3.844 E − 01	20.5667	1.544 E − 01	20.6631	3.377 E − 01	18.8919	1.781	18.8919	1.781	**21.247**	1.991 E − 01
Test-image7	2	13.4063	5.406 E − 02	13.4367	7.327 E − 02	13.4677	7.634 E − 02	13.5164	2.055 E − 01	13.5164	2.055 E − 01	**13.807**	4.152 E − 01
	3	14.4874	7.216 E − 15	14.4891	9.919 E − 03	14.5017	1.428 E − 02	**14.8333**	5.21 E − 01	**14.8333**	5.21 E − 01	14.777	1.552 E − 01
	4	18.9729	1.683	19.9867	8.273 E − 01	19.765	1.279	18.786	1.608	18.786	1.608	**20.605**	1.163 E − 01
	5	20.758	8.438 E − 02	20.8177	2.656 E − 02	20.7976	4.461 E − 02	20.2914	8.201 E − 01	20.2914	8.201 E − 01	**21.242**	2.656 E − 02
Test-image8	2	11.8438	4.3 E − 02	11.8668	5.407 E − 15	11.8668	5.407 E − 15	11.8997	2.446 E − 01	11.8997	2.446 E − 01	**12.104**	5.407 E − 15
	3	**14**	2.296	12.9585	1.22	13.4216	1.869	13.5839	2.066	13.5839	2.066	12.923	2.623
	4	18.9048	1.065	19.0982	2.741 E − 02	19.0763	2.186 E − 04	18.2205	1.208	18.2205	1.208	**19.508**	3.498 E − 02
	5	**20.0727**	1.065	19.877	8.866 E − 01	19.814	8.404 E − 01	19.3334	1.306	19.3334	1.306	19.792	1.149
Test-image9	2	11.9154	4.062 E − 01	11.9839	7.209 E − 15	11.9839	7.209 E − 15	11.8432	2.467 E − 01	11.8432	2.467 E − 01	**12.224**	5.259 E − 01
	3	15.7424	1.018	15.0463	7.528 E − 01	14.9934	1.207	15.4494	9.271 E − 01	15.4494	9.271 E − 01	**16.996**	8.454 E − 01
	4	17.4556	2.445 E − 01	17.5233	2.151 E − 02	16.9955	6.37 E − 01	16.8558	1.324	16.8558	1.324	**17.874**	1.97 E − 02
	5	19.5262	1.338	19.9529	1.017	19.5872	1.487	18.4301	1.413	18.4301	1.413	**20.834**	9.763 E − 01
Test-image10	2	11.4204	9.926 E − 02	11.4037	3.605 E − 15	11.4037	3.605 E − 15	11.5017	2.041 E − 01	11.5017	2.041 E − 01	**11.632**	1.194
	3	14.1162	2.497	16.3872	2.101	**16.4987**	2.06	14.3352	2.483	14.3352	2.483	13.168	2.476
	4	19.115	1.18	19.3449	7.209 E − 15	19.2591	1.747 E − 01	17.5211	2.184	17.5211	2.184	**19.732**	1.137 E − 01
	5	20.4837	7.652 E − 01	20.6416	7.272 E − 01	**20.7048**	7.768 E − 01	18.0473	1.799	18.0473	1.799	20.351	8.192 E − 01

**Table 13:** Comparison of Kapur’s method FSIM values

Test Image		HHO		LSHADE		WOA		SCA		BWOA		IBWOA	
	Th	Mean	Std	Mean	Std	Mean	Std	Mean	Std	Mean	Std	Mean	Std
Test-image1	2	0.852	4.282 E − 03	0.8455	1.03 E − 02	0.8498	3.647 E − 04	0.8451	1.385 E − 03	0.8451	1.385 E − 03	**0.8576**	1.243 E − 02
	3	0.8533	4.038 E − 03	0.8537	3.666 E − 03	0.8532	1.42 E − 03	**0.8556**	8.146 E − 03	**0.8556**	8.146 E − 03	0.8536	2.554 E − 03
	4	0.8726	5.31 E − 03	0.8741	8.544 E − 03	0.8719	9.59 E − 03	0.8668	9.11 E − 03	0.8668	9.11 E − 03	**0.8752**	1.129 E − 03
	5	0.8727	8.079 E − 03	0.8685	7.856 E − 03	0.8674	9.241 E − 03	0.8656	1.022 E − 02	0.8656	1.022 E − 02	**0.8742**	7.195 E − 03
Test-image2	2	0.8554	3.664 E − 04	0.8554	3.661 E − 04	0.8554	3.955 E − 04	**0.856**	1.046 E − 03	**0.856**	1.046 E − 03	0.8554	3.379 E − 16
	3	0.8655	4.51 E − 16	0.8624	3.941 E − 03	0.8635	8.033 E − 03	0.8617	1.165 E − 02	0.8617	1.165 E − 02	**0.8668**	6.594 E − 03
	4	0.8738	9.257 E − 03	0.8774	6.875 E − 03	0.8756	1.068 E − 02	0.8725	9.307 E − 03	0.8725	9.307 E − 03	**0.8808**	2.008 E − 04
	5	0.8684	2.605 E − 03	0.8693	5.433 E − 03	0.8698	1.014 E − 02	**0.8717**	9.549 E − 03	**0.8717**	9.549 E − 03	0.8703	5.07 E − 03
Test-image3	2	**0.7987**	3.02 E − 05	**0.7987**	4.506 E − 16	**0.7987**	3.156 E − 04	0.7985	4.626 E − 04	0.7985	4.626 E − 04	**0.7987**	4.506 E − 16
	3	0.8064	4.278 E − 03	0.804	5.828 E − 03	0.805	9.187 E − 03	0.8104	1.276 E − 02	0.8104	1.276 E − 02	**0.8119**	1.169 E − 02
	4	0.8147	1.096 E − 03	0.8158	2.106 E − 03	0.8152	6.445 E − 03	**0.8208**	5.612 E − 03	**0.8208**	5.612 E − 03	0.8154	1.516 E − 03
	5	0.8231	2.222 E − 03	0.8242	4.898 E − 03	0.8233	4.002 E − 03	0.8225	5.278 E − 03	0.8225	5.278 E − 03	**0.8252**	1.257 E − 03
Test-image4	2	0.8424	5.637 E − 16	0.8424	4.843 E − 07	0.8424	4.838 E − 04	**0.8439**	4.279 E − 03	**0.8439**	4.279 E − 03	0.8424	5.632 E − 16
	3	0.8499	8.324 E − 03	0.8511	1.074 E − 02	**0.8546**	1.08 E − 02	0.8515	8.951 E − 03	0.8515	8.951 E − 03	0.8542	1.025 E − 02
	4	0.8723	7.892 E − 16	0.8756	3.829 E − 04	0.8755	8.442 E − 03	0.8743	7.817 E − 03	0.8743	7.817 E − 03	**0.8757**	1.65 E − 04
	5	0.8857	9.508 E − 03	**0.8931**	9.506 E − 03	0.8899	9.189 E − 03	0.8857	8.806 E − 03	0.8857	8.806 E − 03	0.8911	1 E − 02
Test-image5	2	0.8644	4.51 E − 16	0.8644	4.506 E − 16	0.8644	2.092 E − 03	0.8628	3.48 E − 03	0.8628	3.48 E − 03	**0.8647**	1.816 E − 03
	3	0.8686	1.573 E − 02	0.8696	1.804 E − 02	0.8605	2.201 E − 03	0.8767	3.536 E − 03	0.8767	3.536 E − 03	**0.8788**	1.03 E − 03
	4	0.8676	6.014 E − 04	0.8675	1.707 E − 03	0.8672	3.194 E − 03	0.8661	6.951 E − 03	0.8661	6.951 E − 03	**0.8682**	1.951 E − 03
	5	0.8646	5.006 E − 03	0.8653	4.777 E − 03	0.8639	5.928 E − 03	0.8645	6.547 E − 03	0.8645	6.547 E − 03	**0.869**	2.96 E − 03
Test-image6	2	**0.8336**	5.637 E − 16	0.8335	5.632 E − 16	0.8335	9.537 E − 04	0.8335	1.567 E − 03	0.8335	1.567 E − 03	**0.8336**	3.487 E − 04
	3	0.8453	1.134 E − 03	0.8402	6.982 E − 03	0.8434	5.299 E − 03	0.8443	1.114 E − 02	0.8443	1.114 E − 02	**0.8497**	1.343 E − 02
	4	0.8579	5.201 E − 04	0.8584	7.057 E − 03	0.8561	8.864 E − 03	**0.8634**	1.099 E − 02	**0.8634**	1.099 E − 02	0.8584	3.365 E − 03
	5	0.8605	3.575 E − 03	0.8603	5.991 E − 03	0.8628	1.05 E − 02	0.8572	1.396 E − 02	0.8572	1.396 E − 02	**0.8654**	5.704 E − 03
Test-image7	2	0.857	1.301 E − 02	0.8516	1.369 E − 02	0.8462	4.429 E − 03	0.8497	1.382 E − 02	0.8497	1.382 E − 02	**0.8599**	5.618 E − 03
	3	0.8576	8.943 E − 05	0.8576	1.091 E − 03	**0.8586**	3.379 E − 03	0.8562	3.907 E − 03	0.8562	3.907 E − 03	0.8581	6.747 E − 04
	4	0.8565	6.78 E − 04	0.8556	1.386 E − 03	**0.8573**	3.005 E − 03	0.8544	5.907 E − 03	0.8544	5.907 E − 03	0.8569	1.451 E − 03
	5	0.8673	3.565 E − 04	0.8672	9.188 E − 04	0.8673	5.84 E − 03	0.8576	9.37 E − 03	0.8576	9.37 E − 03	**0.8674**	3.222 E − 04
Test-image8	2	**0.8306**	3.382 E − 16	0.8273	3.379 E − 16	0.8273	4.151 E − 03	0.8257	2.871 E − 03	0.8257	2.871 E − 03	0.829	4.734 E − 03
	3	0.8445	1.035 E − 02	0.8533	1.244 E − 02	0.8519	2.316 E − 03	0.8521	7.143 E − 03	0.8521	7.143 E − 03	**0.8557**	1.384 E − 04
	4	0.8382	1.199 E − 03	0.8389	8.96 E − 05	0.8376	7.655 E − 03	0.8358	1.048 E − 02	0.8358	1.048 E − 02	**0.8392**	1.2 E − 03
	5	0.8334	6.106 E − 03	0.8338	5.584 E − 03	0.834	7.295 E − 03	0.8325	1.349 E − 02	0.8325	1.349 E − 02	**0.8379**	3.339 E − 03
Test-image9	2	**0.8042**	6.765 E − 16	0.8033	6.759 E − 16	0.8033	1.419 E − 02	0.8006	5.216 E − 03	0.8006	5.216 E − 03	0.8037	2.153 E − 03
	3	0.8394	1.251 E − 02	0.827	1.664 E − 02	0.8291	1.997 E − 02	0.8354	1.801 E − 02	0.8354	1.801 E − 02	**0.8458**	1.425 E − 02
	4	0.8336	4.72 E − 04	0.8323	3.435 E − 03	0.8308	1.23 E − 02	0.8265	1.343 E − 02	0.8265	1.343 E − 02	**0.8346**	7.112 E − 03
	5	0.8354	2.285 E − 03	0.8355	6.494 E − 03	0.8328	1.23 E − 02	0.8338	1.157 E − 02	0.8338	1.157 E − 02	**0.8364**	2.615 E − 03
Test-image10	2	0.8277	0	0.8285	0	0.8285	9.125 E − 04	**0.8289**	1.304 E − 03	**0.8289**	1.304 E − 03	**0.8289**	2.267 E − 03
	3	0.8459	4.588 E − 03	0.8393	4.904 E − 03	0.8406	6.627 E − 03	0.8404	1.006 E − 02	0.8404	1.006 E − 02	**0.8465**	6.312 E − 03
	4	0.8539	0	0.8545	4.218 E − 03	0.8535	8.597 E − 03	0.8498	9.258 E − 03	0.8498	9.258 E − 03	**0.8547**	6.452 E − 04
	5	0.8556	3.85 E − 03	0.8556	6.563 E − 03	0.8545	9.589 E − 03	0.8549	1.08 E − 02	0.8549	1.08 E − 02	**0.8583**	3.687 E − 03

Table 10 presents the fitness outcomes received from each level using the proposed IBWOA and other comparison algorithms, based on mean and standard deviation (STD). The most important values are shown in bold. Ideally, the mean should be higher and the STD lower. The proposed IBWOA achieves the highest mean values for different test images compared to other algorithms. Table 11 compares the mean and standard deviation of the Structural Similarity Index Measure (SSIM) for all methods, with optimal findings highlighted in bold, indicating superior image segmentation. Out of forty total experiments, the HHO algorithm yields the best SSIM values four times, LSHADE and SCA each achieve two, and WOA provides three optimal results. The IBWOA method, followed by the BWOA algorithm, consistently obtains the best SSIM values. SSIM is crucial for determining the quality of the structures that remain after segmentation, helping to identify the best approach for segmenting breast thermographic images.

Table 12 shows the PSNR output data. As previously mentioned, PSNR evaluates the similarity between the output image and the source, with higher values indicating better threshold efficiency. It is clear that IBWOA outperforms the original BWOA in terms of PSNR for all test images at each level, surpassing all other tested algorithms. Table 13 presents the mean and standard deviation of the Feature Similarity Index Metric (FSIM) based on Kapur’s entropy as the objective function, with optimal findings denoted in bold. Bold values indicate superior results for quality segmentation. The LSHADE algorithm performed poorly, achieving only two optimal results across all test images and levels. The HHO and WOA algorithms also underperformed, each generating the best results only four times. Among all methods, the proposed IBWOA generates the best FSIM values. The BWOA also identifies better threshold values, yielding expected results with better features using FSIM. This table demonstrates that several methods can work effectively with a limited number of variables.

## Conclusions and future work

In conclusion, breast cancer remains one of the most common cancers, and early detection is crucial for reducing mortality. Thermography offers a cost-effective and suitable screening method compared to mammography, ultrasound, and MRI, as it detects abnormal temperature changes indicative of breast cancer. Effective medical image segmentation is vital for accurate analysis, and thresholding is a key technique in this process. This study presents an innovative approach for determining optimal thresholding values using the Improved Black Widow Optimization Algorithm (IBWOA), which combines quasi-opposite-based learning and the Lévy optimization algorithm to enhance the exploitation phase and avoid local optima. The performance of IBWOA was compared with other techniques, including HHO, LSHADE, WOA, SCA, and BWOA, using Otsu’s and Kapur’s methods on thermal images from the DMR-IR database. The results, evaluated through fitness values, PSNR, SSIM, and FSIM, demonstrated that IBWOA outperforms the other methods. Future work will focus on integrating deep learning methodologies like Convolutional Neural Networks, expanding datasets, and combining multiple imaging modalities to further enhance diagnostic accuracy and applicability in real-world scenarios.

## Data Availability

The dataset for breast cancer is taken from Digital Database for Screening Mammography (DDSM): Breast Cancer Image Dataset [70]. http://visual.ic.uff.br/dmi/prontuario/home.php.
